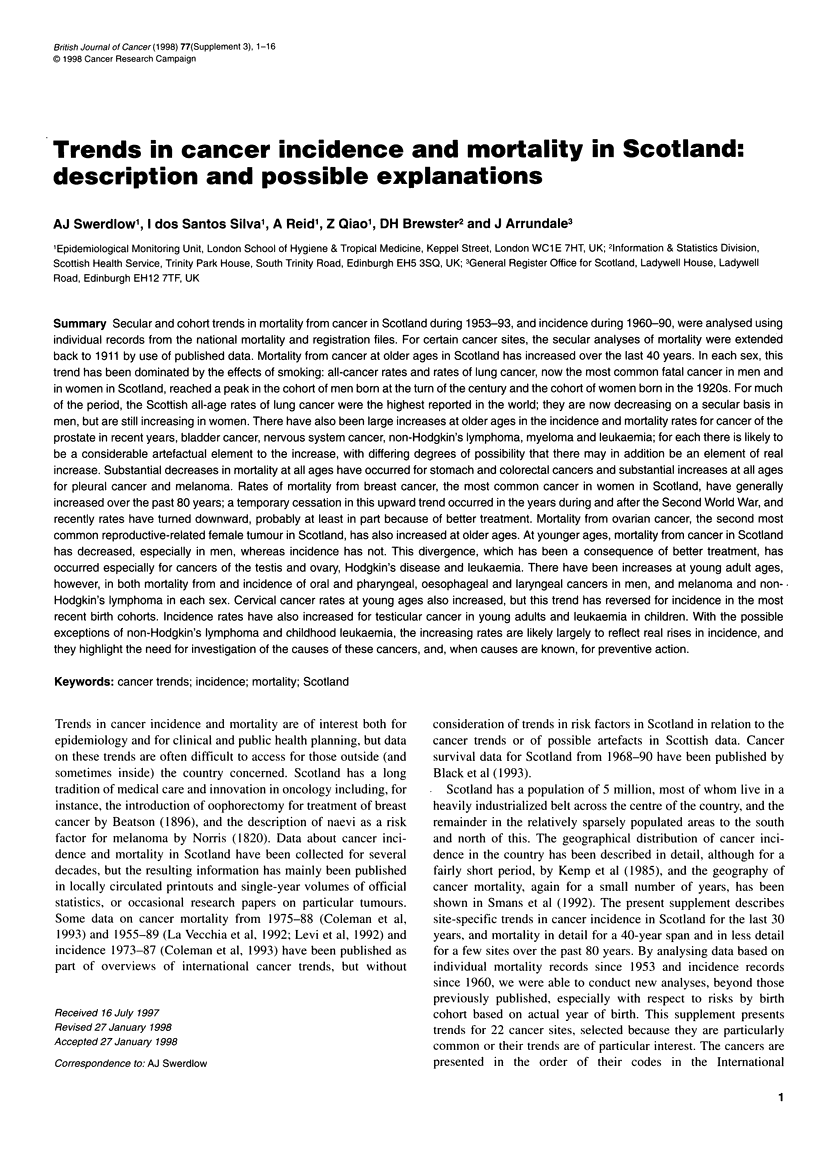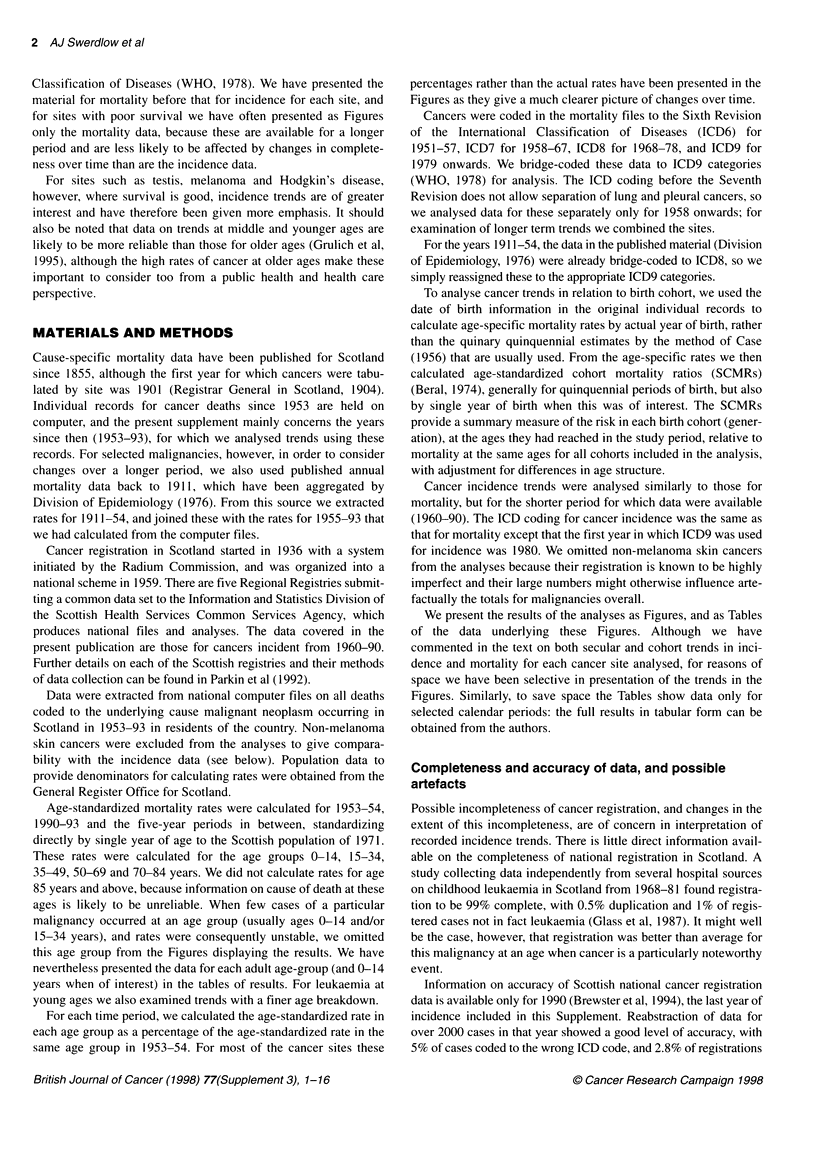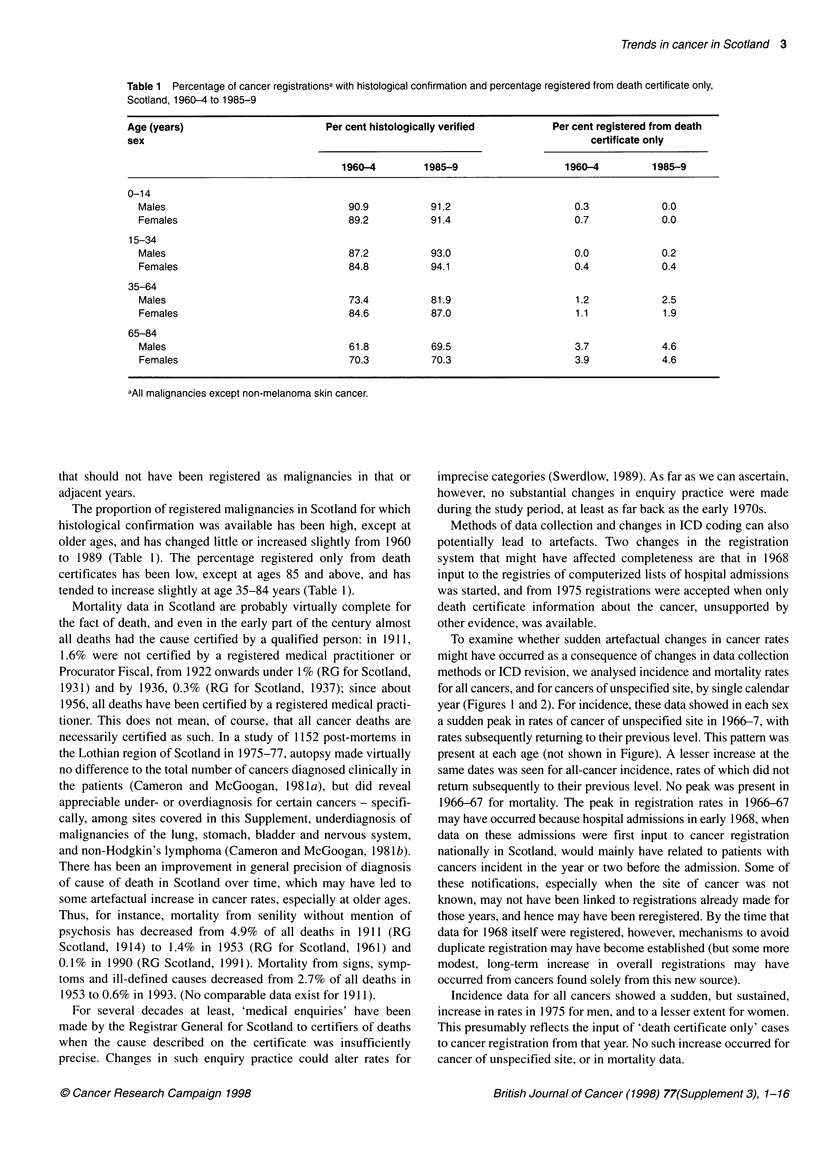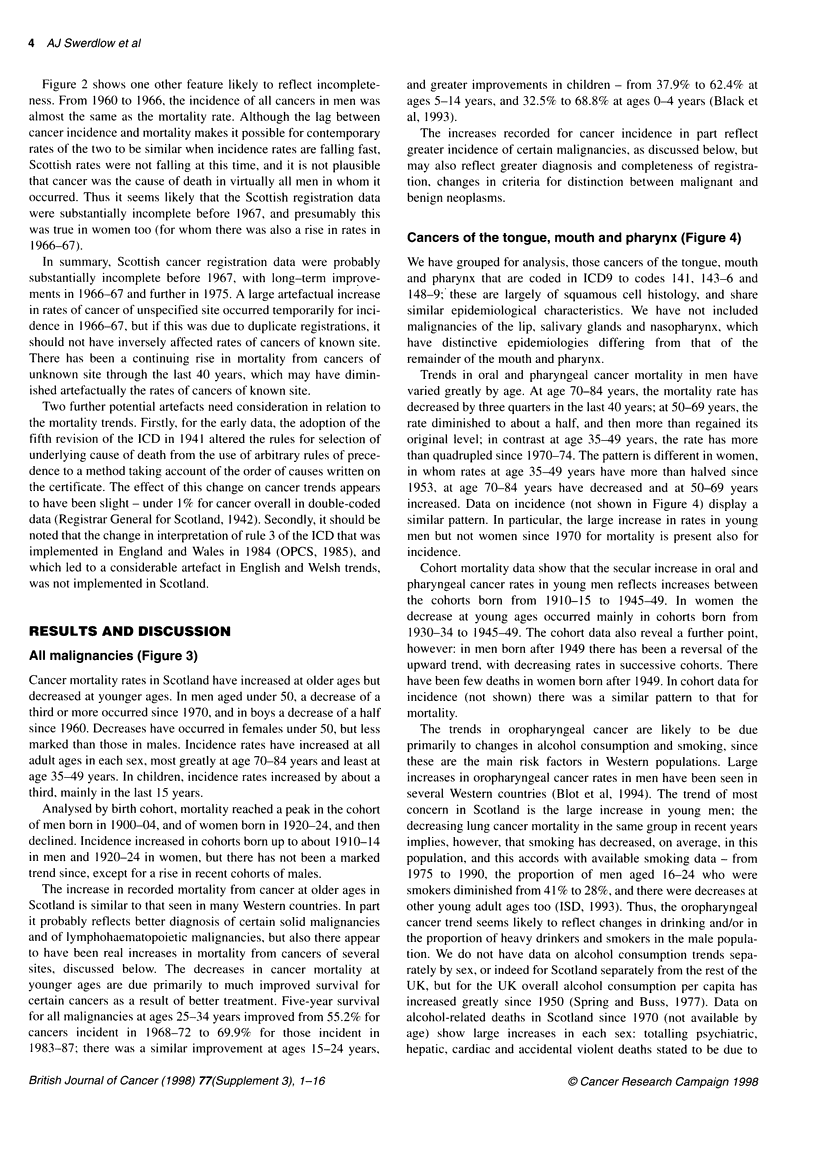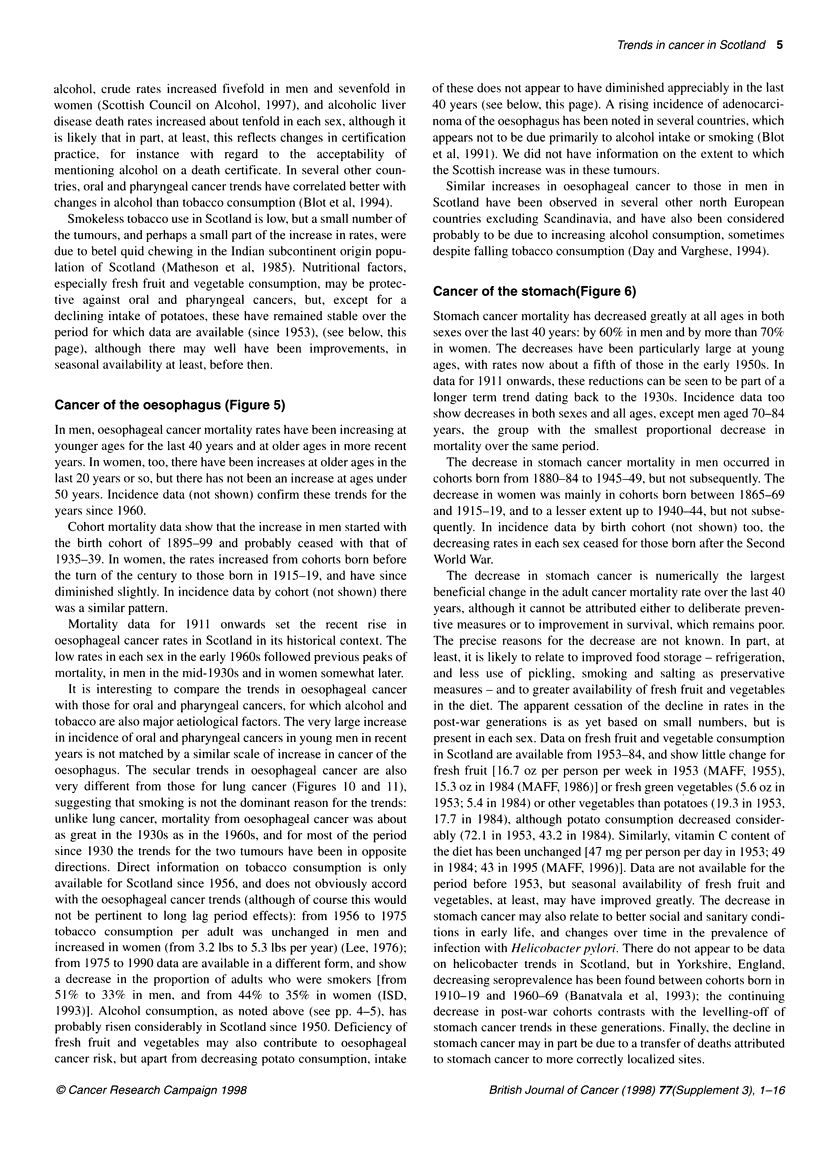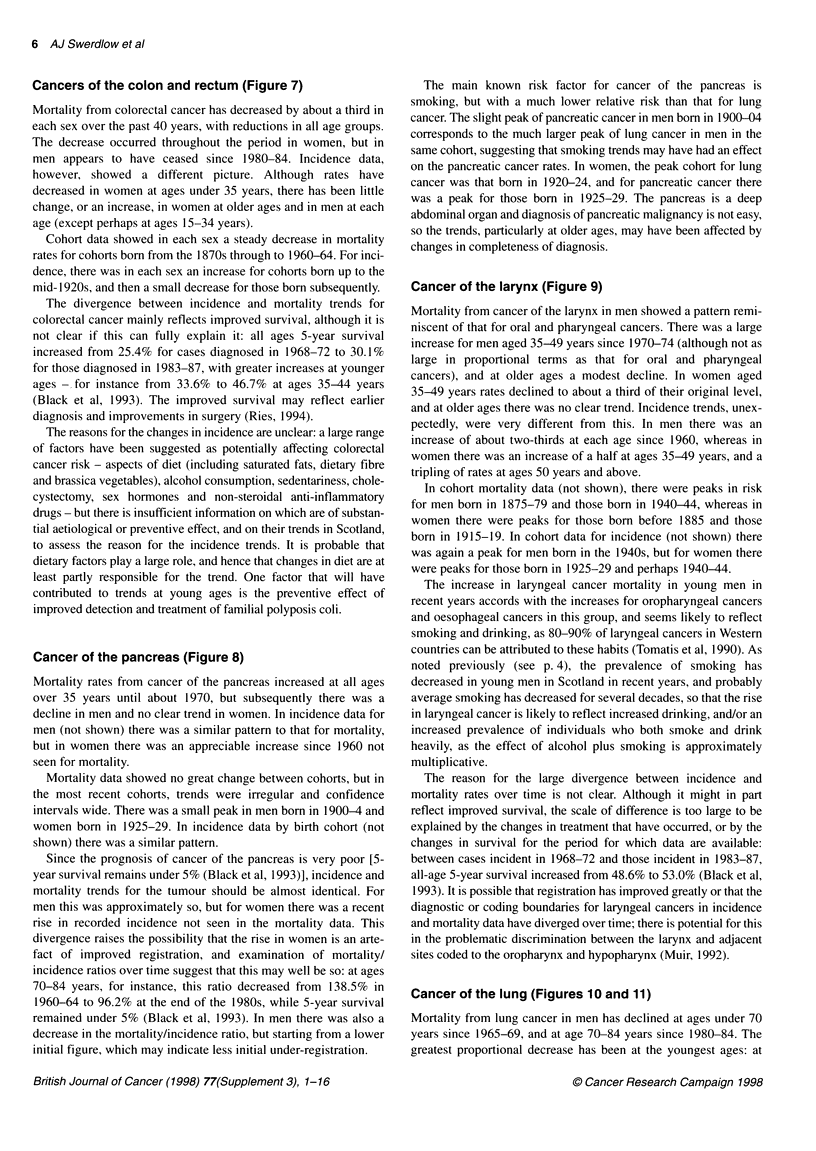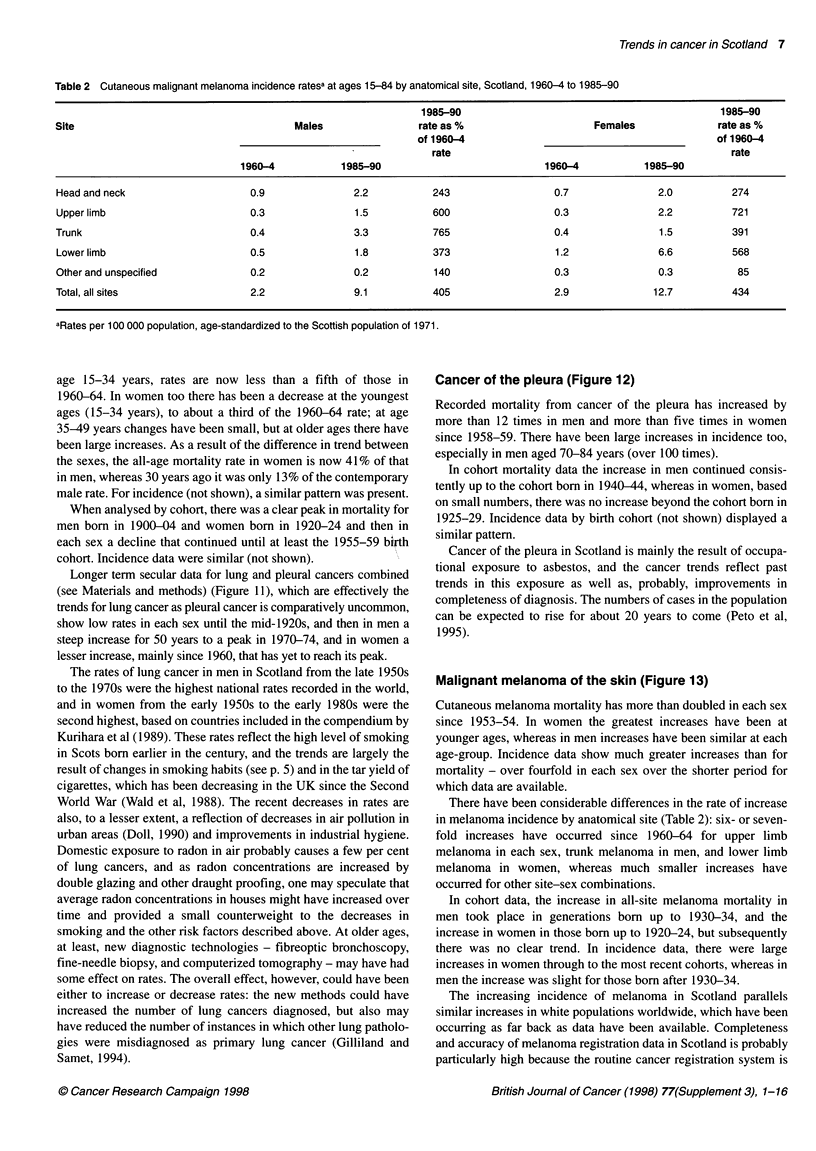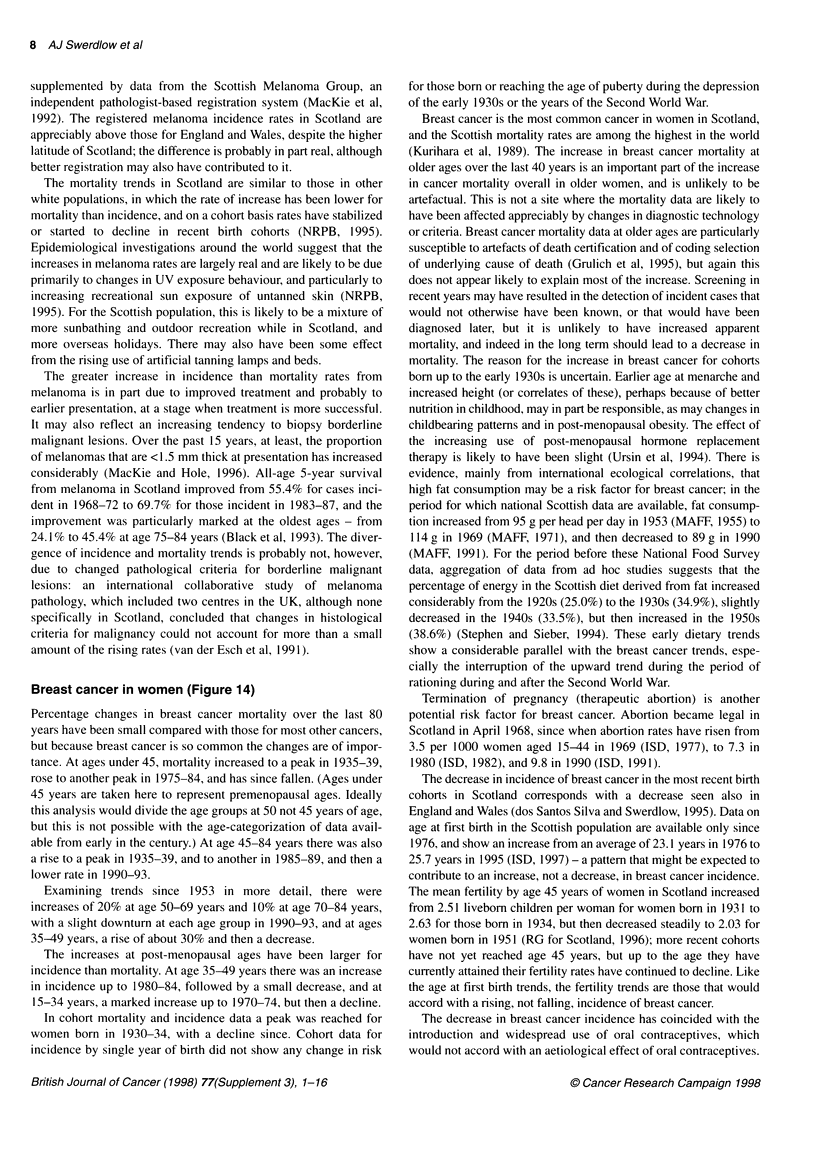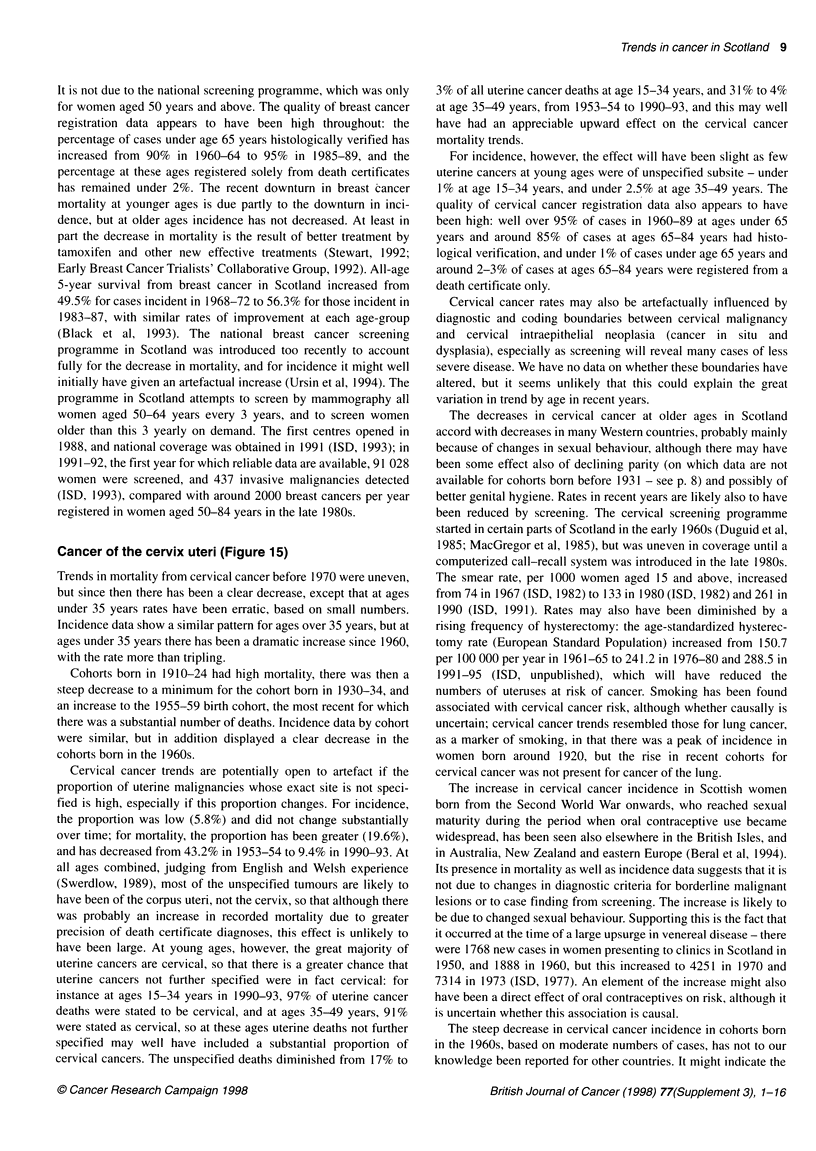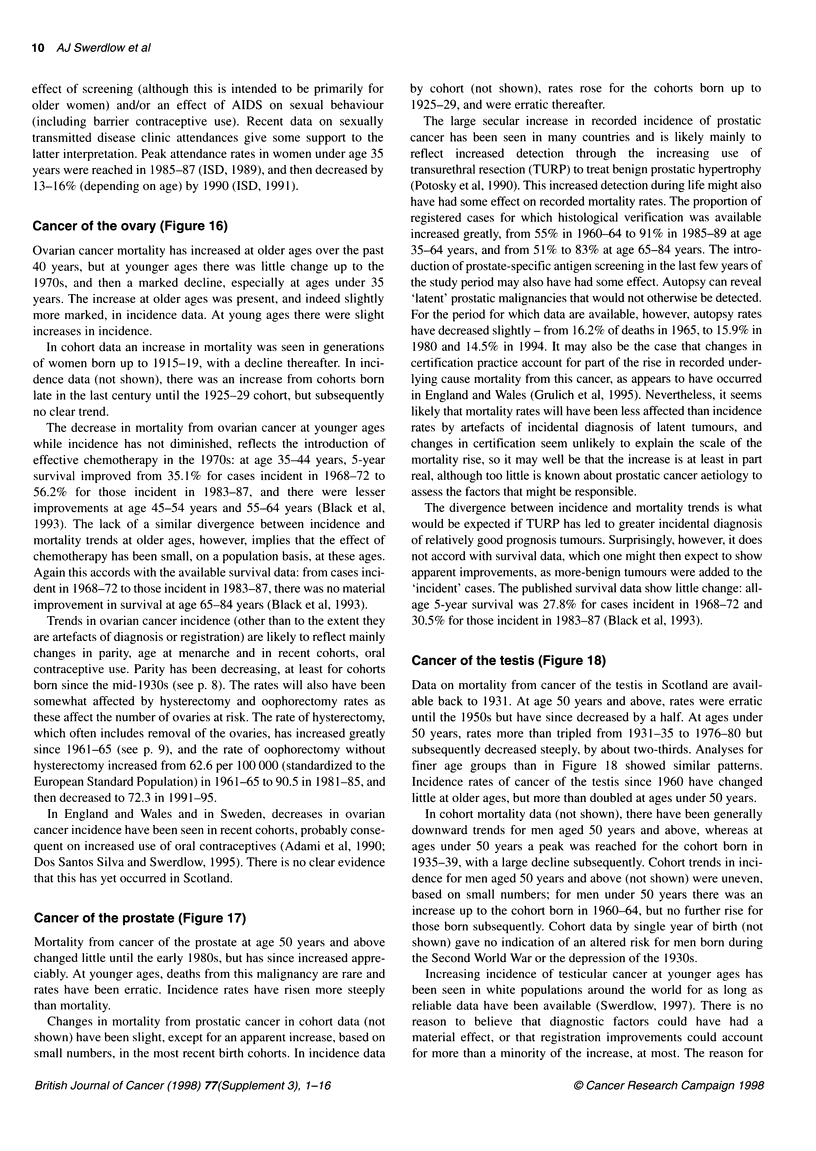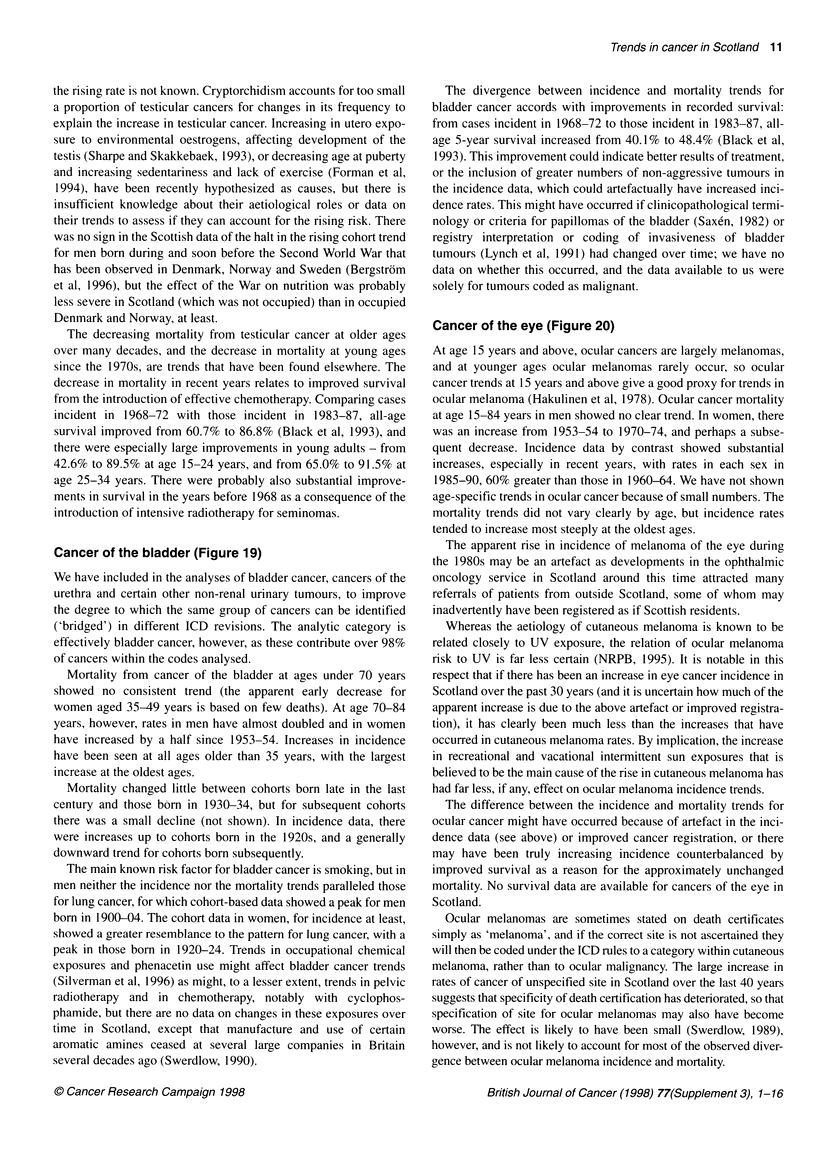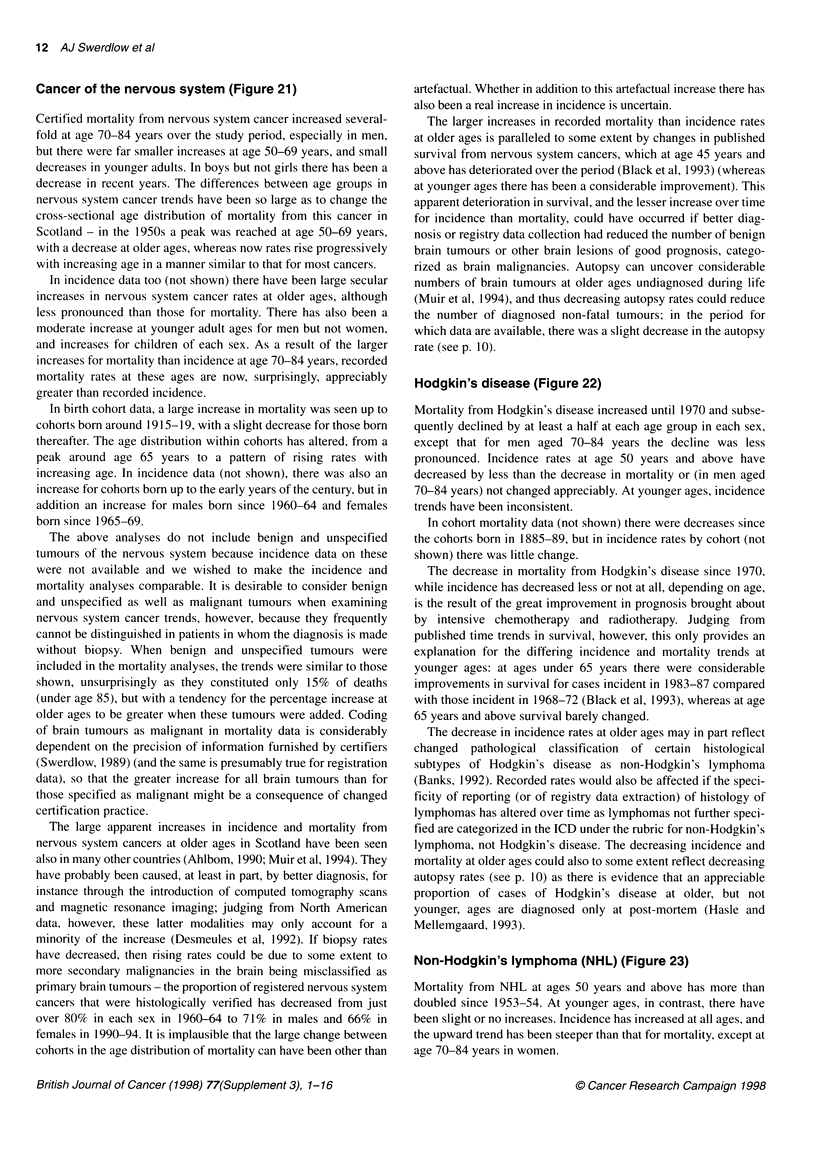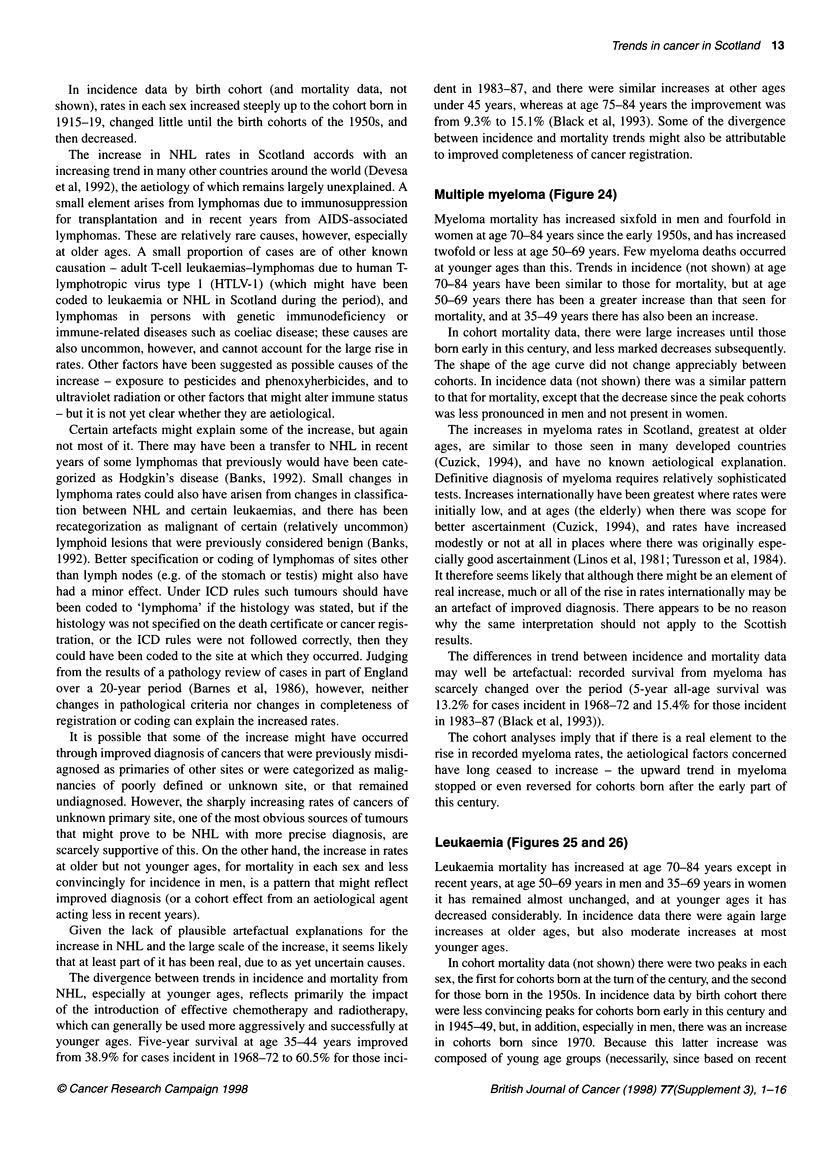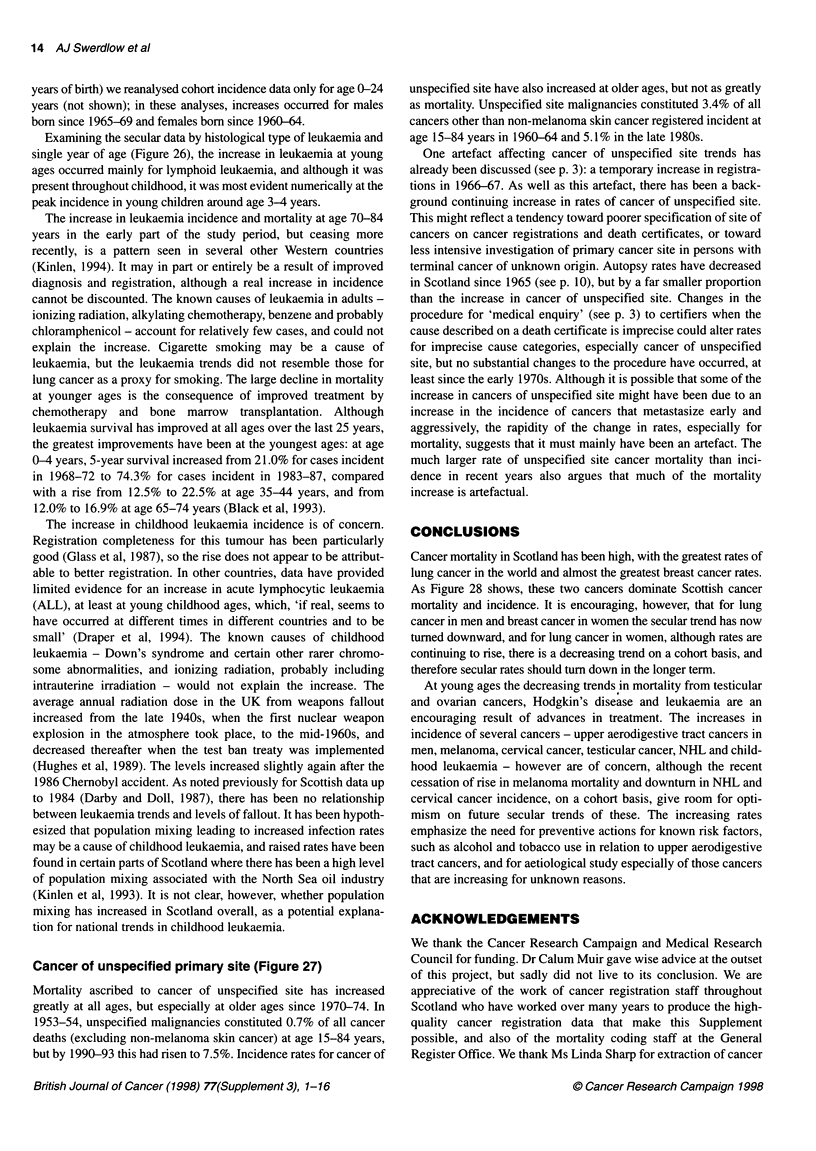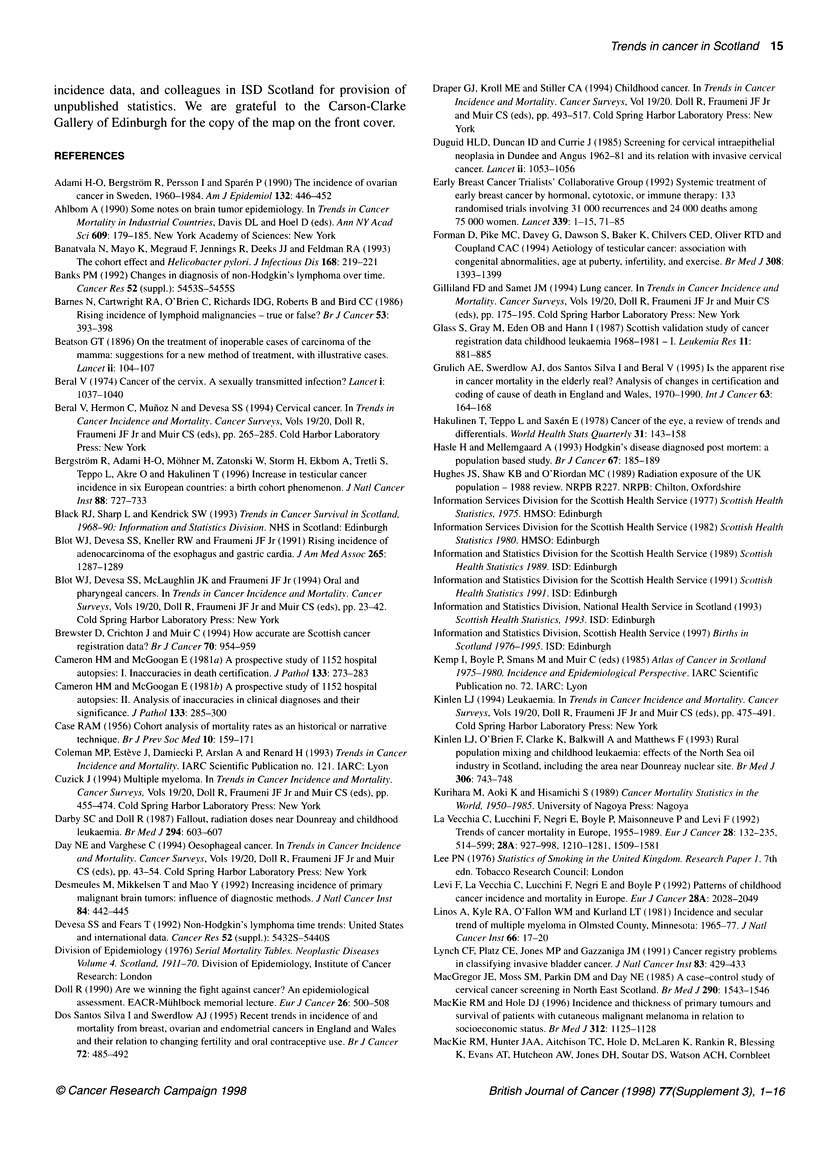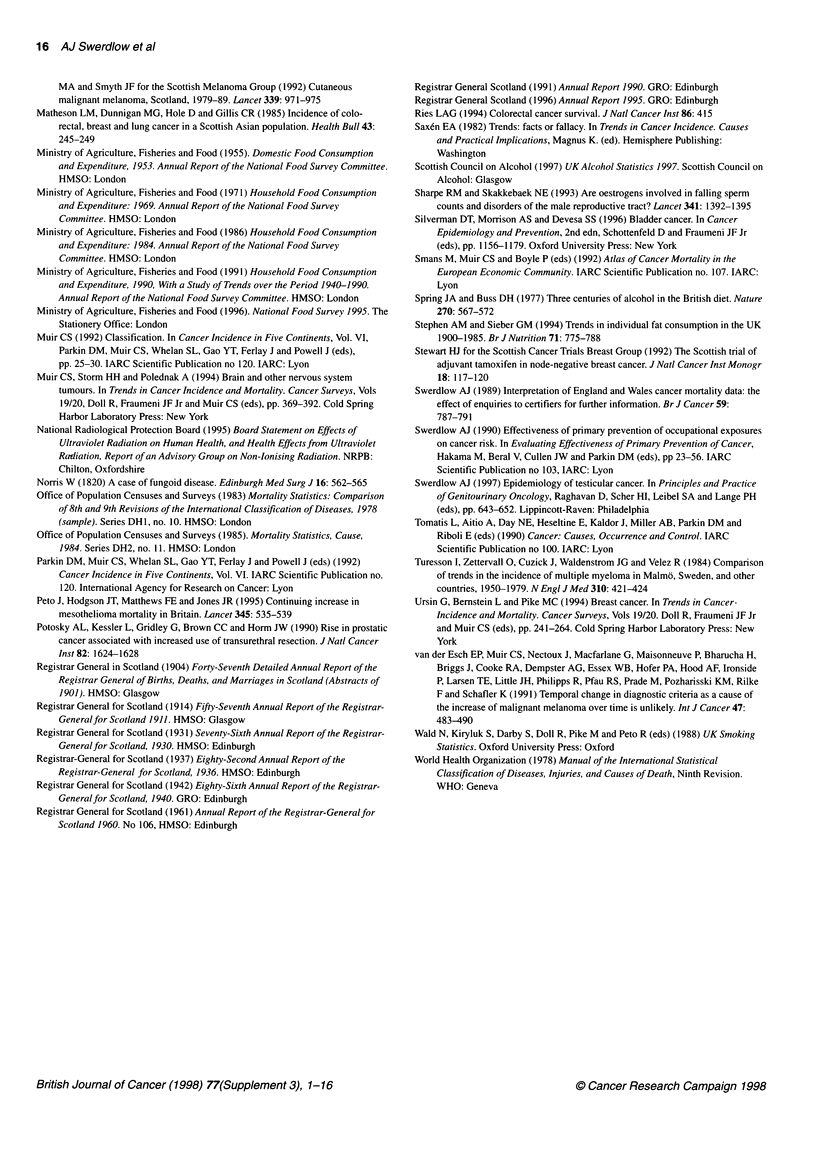# Trends in cancer incidence and mortality in Scotland: description and possible explanations.

**DOI:** 10.1038/bjc.1998.424

**Published:** 1998

**Authors:** A. J. Swerdlow, I. dos Santos Silva, A. Reid, Z. Qiao, D. H. Brewster, J. Arrundale

**Affiliations:** Epidemiological Monitoring Unit, London School of Hygiene & Tropical Medicine, UK.

## Abstract

Secular and cohort trends in mortality from cancer in Scotland during 1953-93, and incidence during 1960-90, were analysed using individual records from the national mortality and registration files. For certain cancer sites, the secular analyses of mortality were extended back to 1911 by use of published data. Mortality from cancer at older ages in Scotland has increased over the last 40 years. In each sex, this trend has been dominated by the effects of smoking: all-cancer rates and rates of lung cancer, now the most common fatal cancer in men and in women in Scotland, reached a peak in the cohort of men born at the turn of the century and the cohort of women born in the 1920s. For much of the period, the Scottish all-age rates of lung cancer were the highest reported in the world; they are now decreasing on a secular basis in men, but are still increasing in women. There have also been large increases at older ages in the incidence and mortality rates for cancer of the prostate in recent years. bladder cancer, nervous system cancer, non-Hodgkin's lymphoma, myeloma and leukaemia; for each there is likely to be a considerable artefactual element to the increase, with differing degrees of possibility that there may in addition be an element of real increase. Substantial decreases in mortality at all ages have occurred for stomach and colorectal cancers and substantial increases at all ages for pleural cancer and melanoma. Rates of mortality from breast cancer, the most common cancer in women in Scotland, have generally increased over the past 80 years; a temporary cessation in this upward trend occurred in the years during and after the Second World War, and recently rates have turned downward, probably at least in part because of better treatment. Mortality from ovarian cancer, the second most common reproductive-related female tumour in Scotland, has also increased at older ages. At younger ages, mortality from cancer in Scotland has decreased, especially in men, whereas incidence has not. This divergence, which has been a consequence of better treatment, has occurred especially for cancers of the testis and ovary, Hodgkin's disease and leukaemia. There have been increases at young adult ages, however, in both mortality from and incidence of oral and pharyngeal, oesophageal and laryngeal cancers in men, and melanoma and non-Hodgkin's lymphoma in each sex. Cervical cancer rates at young ages also increased, but this trend has reversed for incidence in the most recent birth cohorts. Incidence rates have also increased for testicular cancer in young adults and leukaemia in children. With the possible exceptions of non-Hodgkin's lymphoma and childhood leukaemia, the increasing rates are likely largely to reflect real rises in incidence, and they highlight the need for investigation of the causes of these cancers, and, when causes are known, for preventive action.


British Journal of Cancer (1998) 77(Supplement 3), 1–16
© 1998 Cancer Research Campaign

### Trends in cancer incidence and mortality in Scotland: description and possible explanations

**AJ Swerdlow[1], I dos Santos Silva[1], A Reid[1], Z Qiao[1], DH Brewster[2] and J Arrundale[3]**

[1]Epidemiological Monitoring Unit, London School of Hygiene & Tropical Medicine, Keppel Street, London WC1E 7HT, UK; [2]Information & Statistics Division, Scottish Health Service, Trinity Park House, South Trinity Road, Edinburgh EH5 3SQ, UK; [3]General Register Office for Scotland, Ladywell House, Ladywell Road, Edinburgh EH12 7TF, UK

**Summary** Secular and cohort trends in mortality from cancer in Scotland during 1953–93, and incidence during 1960–90, were analysed using individual records from the national mortality and registration files. For certain cancer sites, the secular analyses of mortality were extended back to 1911 by use of published data. Mortality from cancer at older ages in Scotland has increased over the last 40 years. In each sex, this trend has been dominated by the effects of smoking: all-cancer rates and rates of lung cancer, now the most common fatal cancer in men and in women in Scotland, reached a peak in the cohort of men born at the turn of the century and the cohort of women born in the 1920s. For much of the period, the Scottish all-age rates of lung cancer were the highest reported in the world; they are now decreasing on a secular basis in men, but are still increasing in women. There have also been large increases at older ages in the incidence and mortality rates for cancer of the prostate in recent years, bladder cancer, nervous system cancer, non-Hodgkin's lymphoma, myeloma and leukaemia; for each there is likely to be a considerable artefactual element to the increase, with differing degrees of possibility that there may in addition be an element of real increase. Substantial decreases in mortality at all ages have occurred for stomach and colorectal cancers and substantial increases at all ages for pleural cancer and melanoma. Rates of mortality from breast cancer, the most common cancer in women in Scotland, have generally increased over the past 80 years; a temporary cessation in this upward trend occurred in the years during and after the Second World War, and recently rates have turned downward, probably at least in part because of better treatment. Mortality from ovarian cancer, the second most common reproductive-related female tumour in Scotland, has also increased at older ages. At younger ages, mortality from cancer in Scotland has decreased, especially in men, whereas incidence has not. This divergence, which has been a consequence of better treatment, has occurred especially for cancers of the testis and ovary, Hodgkin's disease and leukaemia. There have been increases at young adult ages, however, in both mortality from and incidence of oral and pharyngeal, oesophageal and laryngeal cancers in men, and melanoma and non-Hodgkin's lymphoma in each sex. Cervical cancer rates at young ages also increased, but this trend has reversed for incidence in the most recent birth cohorts. Incidence rates have also increased for testicular cancer in young adults and leukaemia in children. With the possible exceptions of non-Hodgkin's lymphoma and childhood leukaemia, the increasing rates are likely largely to reflect real rises in incidence, and they highlight the need for investigation of the causes of these cancers, and, when causes are known, for preventive action.

**Keywords:** cancer trends; incidence; mortality; Scotland

Trends in cancer incidence and mortality are of interest both for epidemiology and for clinical and public health planning, but data on these trends are often difficult to access for those outside (and sometimes inside) the country concerned. Scotland has a long tradition of medical care and innovation in oncology including, for instance, the introduction of oophorectomy for treatment of breast cancer by Beatson (1896), and the description of naevi as a risk factor for melanoma by Norris (1820). Data about cancer incidence and mortality in Scotland have been collected for several decades, but the resulting information has mainly been published in locally circulated printouts and single-year volumes of official statistics, or occasional research papers on particular tumours. Some data on cancer mortality from 1975–88 (Coleman et al, 1993) and 1955–89 (La Vecchia et al, 1992; Levi et al, 1992) and incidence 1973–87 (Coleman et al, 1993) have been published as part of overviews of international cancer trends, but without consideration of trends in risk factors in Scotland in relation to the cancer trends or of possible artefacts in Scottish data. Cancer survival data for Scotland from 1968–90 have been published by Black et al (1993).

Scotland has a population of 5 million, most of whom live in a heavily industrialized belt across the centre of the country, and the remainder in the relatively sparsely populated areas to the south and north of this. The geographical distribution of cancer incidence in the country has been described in detail, although for a fairly short period, by Kemp et al (1985), and the geography of cancer mortality, again for a small number of years, has been shown in Smans et al (1992). The present supplement describes site-specific trends in cancer incidence in Scotland for the last 30 years, and mortality in detail for a 40-year span and in less detail for a few sites over the past 80 years. By analysing data based on individual mortality records since 1953 and incidence records since 1960, we were able to conduct new analyses, beyond those previously published, especially with respect to risks by birth cohort based on actual year of birth. This supplement presents trends for 22 cancer sites, selected because they are particularly common or their trends are of particular interest. The cancers are presented in the order of their codes in the International

*Received 16 July 1997*
*Revised 27 January 1998*
*Accepted 27 January 1998*

*Correspondence to:* AJ Swerdlow

Classification of Diseases (WHO, 1978). We have presented the material for mortality before that for incidence for each site, and for sites with poor survival we have often presented as Figures only the mortality data, because these are available for a longer period and are less likely to be affected by changes in completeness over time than are the incidence data.

For sites such as testis, melanoma and Hodgkin's disease, however, where survival is good, incidence trends are of greater interest and have therefore been given more emphasis. It should also be noted that data on trends at middle and younger ages are likely to be more reliable than those for older ages (Grulich et al, 1995), although the high rates of cancer at older ages make these important to consider too from a public health and health care perspective.

### MATERIALS AND METHODS

Cause-specific mortality data have been published for Scotland since 1855, although the first year for which cancers were tabulated by site was 1901 (Registrar General in Scotland, 1904). Individual records for cancer deaths since 1953 are held on computer, and the present supplement mainly concerns the years since then (1953–93), for which we analysed trends using these records. For selected malignancies, however, in order to consider changes over a longer period, we also used published annual mortality data back to 1911, which have been aggregated by Division of Epidemiology (1976). From this source we extracted rates for 1911–54, and joined these with the rates for 1955–93 that we had calculated from the computer files.

Cancer registration in Scotland started in 1936 with a system initiated by the Radium Commission, and was organized into a national scheme in 1959. There are five Regional Registries submitting a common data set to the Information and Statistics Division of the Scottish Health Services Common Services Agency, which produces national files and analyses. The data covered in the present publication are those for cancers incident from 1960–90. Further details on each of the Scottish registries and their methods of data collection can be found in Parkin et al (1992).

Data were extracted from national computer files on all deaths coded to the underlying cause malignant neoplasm occurring in Scotland in 1953–93 in residents of the country. Non-melanoma skin cancers were excluded from the analyses to give comparability with the incidence data (see below). Population data to provide denominators for calculating rates were obtained from the General Register Office for Scotland.

Age-standardized mortality rates were calculated for 1953–54, 1990–93 and the five-year periods in between, standardizing directly by single year of age to the Scottish population of 1971. These rates were calculated for the age groups 0–14, 15–34, 35–49, 50–69 and 70–84 years. We did not calculate rates for age 85 years and above, because information on cause of death at these ages is likely to be unreliable. When few cases of a particular malignancy occurred at an age group (usually ages 0–14 and/or 15–34 years), and rates were consequently unstable, we omitted this age group from the Figures displaying the results. We have nevertheless presented the data for each adult age-group (and 0–14 years when of interest) in the tables of results. For leukaemia at young ages we also examined trends with a finer age breakdown.

For each time period, we calculated the age-standardized rate in each age group as a percentage of the age-standardized rate in the same age group in 1953–54. For most of the cancer sites these percentages rather than the actual rates have been presented in the Figures as they give a much clearer picture of changes over time.

Cancers were coded in the mortality files to the Sixth Revision of the International Classification of Diseases (ICD6) for 1951–57, ICD7 for 1958–67, ICD8 for 1968–78, and ICD9 for 1979 onwards. We bridge-coded these data to ICD9 categories (WHO, 1978) for analysis. The ICD coding before the Seventh Revision does not allow separation of lung and pleural cancers, so we analysed data for these separately only for 1958 onwards; for examination of longer term trends we combined the sites.

For the years 1911–54, the data in the published material (Division of Epidemiology, 1976) were already bridge-coded to ICD8, so we simply reassigned these to the appropriate ICD9 categories.

To analyse cancer trends in relation to birth cohort, we used the date of birth information in the original individual records to calculate age-specific mortality rates by actual year of birth, rather than the quinary quinquennial estimates by the method of Case (1956) that are usually used. From the age-specific rates we then calculated age-standardized cohort mortality ratios (SCMRs) (Beral, 1974), generally for quinquennial periods of birth, but also by single year of birth when this was of interest. The SCMRs provide a summary measure of the risk in each birth cohort (generation), at the ages they had reached in the study period, relative to mortality at the same ages for all cohorts included in the analysis, with adjustment for differences in age structure.

Cancer incidence trends were analysed similarly to those for mortality, but for the shorter period for which data were available (1960–90). The ICD coding for cancer incidence was the same as that for mortality except that the first year in which ICD9 was used for incidence was 1980. We omitted non-melanoma skin cancers from the analyses because their registration is known to be highly imperfect and their large numbers might otherwise influence artefactually the totals for malignancies overall.

We present the results of the analyses as Figures, and as Tables of the data underlying these Figures. Although we have commented in the text on both secular and cohort trends in incidence and mortality for each cancer site analysed, for reasons of space we have been selective in presentation of the trends in the Figures. Similarly, to save space the Tables show data only for selected calendar periods: the full results in tabular form can be obtained from the authors.

### Completeness and accuracy of data, and possible artefacts

Possible incompleteness of cancer registration, and changes in the extent of this incompleteness, are of concern in interpretation of recorded incidence trends. There is little direct information available on the completeness of national registration in Scotland. A study collecting data independently from several hospital sources on childhood leukaemia in Scotland from 1968–81 found registration to be 99% complete, with 0.5% duplication and 1% of registered cases not in fact leukaemia (Glass et al, 1987). It might well be the case, however, that registration was better than average for this malignancy at an age when cancer is a particularly noteworthy event.

Information on accuracy of Scottish national cancer registration data is available only for 1990 (Brewster et al, 1994), the last year of incidence included in this Supplement. Reabstraction of data for over 2000 cases in that year showed a good level of accuracy, with 5% of cases coded to the wrong ICD code, and 2.8% of registrations

 © Cancer Research Campaign 1998

**Table 1** Percentage of cancer registrations[a] with histological confirmation and percentage registered from death certificate only, Scotland, 1960–4 to 1985–9

| Age (years) sex | Per cent histologically verified | | Per cent registered from death certificate only | |
|---|---|---|---|---|
| | 1960–4 | 1985–9 | 1960–4 | 1985–9 |
| 0–14 | | | | |
| Males | 90.9 | 91.2 | 0.3 | 0.0 |
| Females | 89.2 | 91.4 | 0.7 | 0.0 |
| 15–34 | | | | |
| Males | 87.2 | 93.0 | 0.0 | 0.2 |
| Females | 84.8 | 94.1 | 0.4 | 0.4 |
| 35–64 | | | | |
| Males | 73.4 | 81.9 | 1.2 | 2.5 |
| Females | 84.6 | 87.0 | 1.1 | 1.9 |
| 65–84 | | | | |
| Males | 61.8 | 69.5 | 3.7 | 4.6 |
| Females | 70.3 | 70.3 | 3.9 | 4.6 |

[a]All malignancies except non-melanoma skin cancer.

that should not have been registered as malignancies in that or adjacent years.

The proportion of registered malignancies in Scotland for which histological confirmation was available has been high, except at older ages, and has changed little or increased slightly from 1960 to 1989 (Table 1). The percentage registered only from death certificates has been low, except at ages 85 and above, and has tended to increase slightly at age 35–84 years (Table 1).

Mortality data in Scotland are probably virtually complete for the fact of death, and even in the early part of the century almost all deaths had the cause certified by a qualified person: in 1911, 1.6% were not certified by a registered medical practitioner or Procurator Fiscal, from 1922 onwards under 1% (RG for Scotland, 1931) and by 1936, 0.3% (RG for Scotland, 1937); since about 1956, all deaths have been certified by a registered medical practitioner. This does not mean, of course, that all cancer deaths are necessarily certified as such. In a study of 1152 post-mortems in the Lothian region of Scotland in 1975–77, autopsy made virtually no difference to the total number of cancers diagnosed clinically in the patients (Cameron and McGoogan, 1981*a*), but did reveal appreciable under- or overdiagnosis for certain cancers – specifically, among sites covered in this Supplement, underdiagnosis of malignancies of the lung, stomach, bladder and nervous system, and non-Hodgkin's lymphoma (Cameron and McGoogan, 1981*b*). There has been an improvement in general precision of diagnosis of cause of death in Scotland over time, which may have led to some artefactual increase in cancer rates, especially at older ages. Thus, for instance, mortality from senility without mention of psychosis has decreased from 4.9% of all deaths in 1911 (RG Scotland, 1914) to 1.4% in 1953 (RG for Scotland, 1961) and 0.1% in 1990 (RG Scotland, 1991). Mortality from signs, symptoms and ill-defined causes decreased from 2.7% of all deaths in 1953 to 0.6% in 1993. (No comparable data exist for 1911).

For several decades at least, 'medical enquiries' have been made by the Registrar General for Scotland to certifiers of deaths when the cause described on the certificate was insufficiently precise. Changes in such enquiry practice could alter rates for imprecise categories (Swerdlow, 1989). As far as we can ascertain, however, no substantial changes in enquiry practice were made during the study period, at least as far back as the early 1970s.

Methods of data collection and changes in ICD coding can also potentially lead to artefacts. Two changes in the registration system that might have affected completeness are that in 1968 input to the registries of computerized lists of hospital admissions was started, and from 1975 registrations were accepted when only death certificate information about the cancer, unsupported by other evidence, was available.

To examine whether sudden artefactual changes in cancer rates might have occurred as a consequence of changes in data collection methods or ICD revision, we analysed incidence and mortality rates for all cancers, and for cancers of unspecified site, by single calendar year (Figures 1 and 2). For incidence, these data showed in each sex a sudden peak in rates of cancer of unspecified site in 1966–7, with rates subsequently returning to their previous level. This pattern was present at each age (not shown in Figure). A lesser increase at the same dates was seen for all-cancer incidence, rates of which did not return subsequently to their previous level. No peak was present in 1966–67 for mortality. The peak in registration rates in 1966–67 may have occurred because hospital admissions in early 1968, when data on these admissions were first input to cancer registration nationally in Scotland, would mainly have related to patients with cancers incident in the year or two before the admission. Some of these notifications, especially when the site of cancer was not known, may not have been linked to registrations already made for those years, and hence may have been reregistered. By the time that data for 1968 itself were registered, however, mechanisms to avoid duplicate registration may have become established (but some more modest, long-term increase in overall registrations may have occurred from cancers found solely from this new source).

Incidence data for all cancers showed a sudden, but sustained, increase in rates in 1975 for men, and to a lesser extent for women. This presumably reflects the input of 'death certificate only' cases to cancer registration from that year. No such increase occurred for cancer of unspecified site, or in mortality data.

*© Cancer Research Campaign 1998*

Figure 2 shows one other feature likely to reflect incompleteness. From 1960 to 1966, the incidence of all cancers in men was almost the same as the mortality rate. Although the lag between cancer incidence and mortality makes it possible for contemporary rates of the two to be similar when incidence rates are falling fast, Scottish rates were not falling at this time, and it is not plausible that cancer was the cause of death in virtually all men in whom it occurred. Thus it seems likely that the Scottish registration data were substantially incomplete before 1967, and presumably this was true in women too (for whom there was also a rise in rates in 1966–67).

In summary, Scottish cancer registration data were probably substantially incomplete before 1967, with long-term improvements in 1966–67 and further in 1975. A large artefactual increase in rates of cancer of unspecified site occurred temporarily for incidence in 1966–67, but if this was due to duplicate registrations, it should not have inversely affected rates of cancers of known site. There has been a continuing rise in mortality from cancers of unknown site through the last 40 years, which may have diminished artefactually the rates of cancers of known site.

Two further potential artefacts need consideration in relation to the mortality trends. Firstly, for the early data, the adoption of the fifth revision of the ICD in 1941 altered the rules for selection of underlying cause of death from the use of arbitrary rules of precedence to a method taking account of the order of causes written on the certificate. The effect of this change on cancer trends appears to have been slight – under 1% for cancer overall in double-coded data (Registrar General for Scotland, 1942). Secondly, it should be noted that the change in interpretation of rule 3 of the ICD that was implemented in England and Wales in 1984 (OPCS, 1985), and which led to a considerable artefact in English and Welsh trends, was not implemented in Scotland.

### RESULTS AND DISCUSSION

### All malignancies (Figure 3)

Cancer mortality rates in Scotland have increased at older ages but decreased at younger ages. In men aged under 50, a decrease of a third or more occurred since 1970, and in boys a decrease of a half since 1960. Decreases have occurred in females under 50, but less marked than those in males. Incidence rates have increased at all adult ages in each sex, most greatly at age 70–84 years and least at age 35–49 years. In children, incidence rates increased by about a third, mainly in the last 15 years.

Analysed by birth cohort, mortality reached a peak in the cohort of men born in 1900–04, and of women born in 1920–24, and then declined. Incidence increased in cohorts born up to about 1910–14 in men and 1920–24 in women, but there has not been a marked trend since, except for a rise in recent cohorts of males.

The increase in recorded mortality from cancer at older ages in Scotland is similar to that seen in many Western countries. In part it probably reflects better diagnosis of certain solid malignancies and of lymphohaematopoietic malignancies, but also there appear to have been real increases in mortality from cancers of several sites, discussed below. The decreases in cancer mortality at younger ages are due primarily to much improved survival for certain cancers as a result of better treatment. Five-year survival for all malignancies at ages 25–34 years improved from 55.2% for cancers incident in 1968–72 to 69.9% for those incident in 1983–87; there was a similar improvement at ages 15–24 years, and greater improvements in children – from 37.9% to 62.4% at ages 5–14 years, and 32.5% to 68.8% at ages 0–4 years (Black et al, 1993).

The increases recorded for cancer incidence in part reflect greater incidence of certain malignancies, as discussed below, but may also reflect greater diagnosis and completeness of registration, changes in criteria for distinction between malignant and benign neoplasms.

### Cancers of the tongue, mouth and pharynx (Figure 4)

We have grouped for analysis, those cancers of the tongue, mouth and pharynx that are coded in ICD9 to codes 141, 143–6 and 148–9; these are largely of squamous cell histology, and share similar epidemiological characteristics. We have not included malignancies of the lip, salivary glands and nasopharynx, which have distinctive epidemiologies differing from that of the remainder of the mouth and pharynx.

Trends in oral and pharyngeal cancer mortality in men have varied greatly by age. At age 70–84 years, the mortality rate has decreased by three quarters in the last 40 years; at 50–69 years, the rate diminished to about a half, and then more than regained its original level; in contrast at age 35–49 years, the rate has more than quadrupled since 1970–74. The pattern is different in women, in whom rates at age 35–49 years have more than halved since 1953, at age 70–84 years have decreased and at 50–69 years increased. Data on incidence (not shown in Figure 4) display a similar pattern. In particular, the large increase in rates in young men but not women since 1970 for mortality is present also for incidence.

Cohort mortality data show that the secular increase in oral and pharyngeal cancer rates in young men reflects increases between the cohorts born from 1910–15 to 1945–49. In women the decrease at young ages occurred mainly in cohorts born from 1930–34 to 1945–49. The cohort data also reveal a further point, however: in men born after 1949 there has been a reversal of the upward trend, with decreasing rates in successive cohorts. There have been few deaths in women born after 1949. In cohort data for incidence (not shown) there was a similar pattern to that for mortality.

The trends in oropharyngeal cancer are likely to be due primarily to changes in alcohol consumption and smoking, since these are the main risk factors in Western populations. Large increases in oropharyngeal cancer rates in men have been seen in several Western countries (Blot et al, 1994). The trend of most concern in Scotland is the large increase in young men; the decreasing lung cancer mortality in the same group in recent years implies, however, that smoking has decreased, on average, in this population, and this accords with available smoking data – from 1975 to 1990, the proportion of men aged 16–24 who were smokers diminished from 41% to 28%, and there were decreases at other young adult ages too (ISD, 1993). Thus, the oropharyngeal cancer trend seems likely to reflect changes in drinking and/or in the proportion of heavy drinkers and smokers in the male population. We do not have data on alcohol consumption trends separately by sex, or indeed for Scotland separately from the rest of the UK, but for the UK overall alcohol consumption per capita has increased greatly since 1950 (Spring and Buss, 1977). Data on alcohol-related deaths in Scotland since 1970 (not available by age) show large increases in each sex: totalling psychiatric, hepatic, cardiac and accidental violent deaths stated to be due to

© Cancer Research Campaign 1998

alcohol, crude rates increased fivefold in men and sevenfold in women (Scottish Council on Alcohol, 1997), and alcoholic liver disease death rates increased about tenfold in each sex, although it is likely that in part, at least, this reflects changes in certification practice, for instance with regard to the acceptability of mentioning alcohol on a death certificate. In several other countries, oral and pharyngeal cancer trends have correlated better with changes in alcohol than tobacco consumption (Blot et al, 1994).

Smokeless tobacco use in Scotland is low, but a small number of the tumours, and perhaps a small part of the increase in rates, were due to betel quid chewing in the Indian subcontinent origin population of Scotland (Matheson et al, 1985). Nutritional factors, especially fresh fruit and vegetable consumption, may be protective against oral and pharyngeal cancers, but, except for a declining intake of potatoes, these have remained stable over the period for which data are available (since 1953), (see below, this page), although there may well have been improvements, in seasonal availability at least, before then.

### Cancer of the oesophagus (Figure 5)

In men, oesophageal cancer mortality rates have been increasing at younger ages for the last 40 years and at older ages in more recent years. In women, too, there have been increases at older ages in the last 20 years or so, but there has not been an increase at ages under 50 years. Incidence data (not shown) confirm these trends for the years since 1960.

Cohort mortality data show that the increase in men started with the birth cohort of 1895–99 and probably ceased with that of 1935–39. In women, the rates increased from cohorts born before the turn of the century to those born in 1915–19, and have since diminished slightly. In incidence data by cohort (not shown) there was a similar pattern.

Mortality data for 1911 onwards set the recent rise in oesophageal cancer rates in Scotland in its historical context. The low rates in each sex in the early 1960s followed previous peaks of mortality, in men in the mid-1930s and in women somewhat later.

It is interesting to compare the trends in oesophageal cancer with those for oral and pharyngeal cancers, for which alcohol and tobacco are also major aetiological factors. The very large increase in incidence of oral and pharyngeal cancers in young men in recent years is not matched by a similar scale of increase in cancer of the oesophagus. The secular trends in oesophageal cancer are also very different from those for lung cancer (Figures 10 and 11), suggesting that smoking is not the dominant reason for the trends: unlike lung cancer, mortality from oesophageal cancer was about as great in the 1930s as in the 1960s, and for most of the period since 1930 the trends for the two tumours have been in opposite directions. Direct information on tobacco consumption is only available for Scotland since 1956, and does not obviously accord with the oesophageal cancer trends (although of course this would not be pertinent to long lag period effects): from 1956 to 1975 tobacco consumption per adult was unchanged in men and increased in women (from 3.2 lbs to 5.3 lbs per year) (Lee, 1976); from 1975 to 1990 data are available in a different form, and show a decrease in the proportion of adults who were smokers [from 51% to 33% in men, and from 44% to 35% in women (ISD, 1993)]. Alcohol consumption, as noted above (see pp. 4–5), has probably risen considerably in Scotland since 1950. Deficiency of fresh fruit and vegetables may also contribute to oesophageal cancer risk, but apart from decreasing potato consumption, intake of these does not appear to have diminished appreciably in the last 40 years (see below, this page). A rising incidence of adenocarcinoma of the oesophagus has been noted in several countries, which appears not to be due primarily to alcohol intake or smoking (Blot et al, 1991). We did not have information on the extent to which the Scottish increase was in these tumours.

Similar increases in oesophageal cancer to those in men in Scotland have been observed in several other north European countries excluding Scandinavia, and have also been considered probably to be due to increasing alcohol consumption, sometimes despite falling tobacco consumption (Day and Varghese, 1994).

### Cancer of the stomach(Figure 6)

Stomach cancer mortality has decreased greatly at all ages in both sexes over the last 40 years: by 60% in men and by more than 70% in women. The decreases have been particularly large at young ages, with rates now about a fifth of those in the early 1950s. In data for 1911 onwards, these reductions can be seen to be part of a longer term trend dating back to the 1930s. Incidence data too show decreases in both sexes and all ages, except men aged 70–84 years, the group with the smallest proportional decrease in mortality over the same period.

The decrease in stomach cancer mortality in men occurred in cohorts born from 1880–84 to 1945–49, but not subsequently. The decrease in women was mainly in cohorts born between 1865–69 and 1915–19, and to a lesser extent up to 1940–44, but not subsequently. In incidence data by birth cohort (not shown) too, the decreasing rates in each sex ceased for those born after the Second World War.

The decrease in stomach cancer is numerically the largest beneficial change in the adult cancer mortality rate over the last 40 years, although it cannot be attributed either to deliberate preventive measures or to improvement in survival, which remains poor. The precise reasons for the decrease are not known. In part, at least, it is likely to relate to improved food storage – refrigeration, and less use of pickling, smoking and salting as preservative measures – and to greater availability of fresh fruit and vegetables in the diet. The apparent cessation of the decline in rates in the post-war generations is as yet based on small numbers, but is present in each sex. Data on fresh fruit and vegetable consumption in Scotland are available from 1953–84, and show little change for fresh fruit [16.7 oz per person per week in 1953 (MAFF, 1955), 15.3 oz in 1984 (MAFF, 1986)] or fresh green vegetables (5.6 oz in 1953; 5.4 in 1984) or other vegetables than potatoes (19.3 in 1953, 17.7 in 1984), although potato consumption decreased considerably (72.1 in 1953, 43.2 in 1984). Similarly, vitamin C content of the diet has been unchanged [47 mg per person per day in 1953; 49 in 1984; 43 in 1995 (MAFF, 1996)]. Data are not available for the period before 1953, but seasonal availability of fresh fruit and vegetables, at least, may have improved greatly. The decrease in stomach cancer may also relate to better social and sanitary conditions in early life, and changes over time in the prevalence of infection with *Helicobacter pylori*. There do not appear to be data on helicobacter trends in Scotland, but in Yorkshire, England, decreasing seroprevalence has been found between cohorts born in 1910–19 and 1960–69 (Banatvala et al, 1993); the continuing decrease in post-war cohorts contrasts with the levelling-off of stomach cancer trends in these generations. Finally, the decline in stomach cancer may in part be due to a transfer of deaths attributed to stomach cancer to more correctly localized sites.

© Cancer Research Campaign 1998

### Cancers of the colon and rectum (Figure 7)

Mortality from colorectal cancer has decreased by about a third in each sex over the past 40 years, with reductions in all age groups. The decrease occurred throughout the period in women, but in men appears to have ceased since 1980–84. Incidence data, however, showed a different picture. Although rates have decreased in women at ages under 35 years, there has been little change, or an increase, in women at older ages and in men at each age (except perhaps at ages 15–34 years).

Cohort data showed in each sex a steady decrease in mortality rates for cohorts born from the 1870s through to 1960–64. For incidence, there was in each sex an increase for cohorts born up to the mid-1920s, and then a small decrease for those born subsequently.

The divergence between incidence and mortality trends for colorectal cancer mainly reflects improved survival, although it is not clear if this can fully explain it: all ages 5-year survival increased from 25.4% for cases diagnosed in 1968–72 to 30.1% for those diagnosed in 1983–87, with greater increases at younger ages – for instance from 33.6% to 46.7% at ages 35–44 years (Black et al, 1993). The improved survival may reflect earlier diagnosis and improvements in surgery (Ries, 1994).

The reasons for the changes in incidence are unclear: a large range of factors have been suggested as potentially affecting colorectal cancer risk – aspects of diet (including saturated fats, dietary fibre and brassica vegetables), alcohol consumption, sedentariness, cholecystectomy, sex hormones and non-steroidal anti-inflammatory drugs – but there is insufficient information on which are of substantial aetiological or preventive effect, and on their trends in Scotland, to assess the reason for the incidence trends. It is probable that dietary factors play a large role, and hence that changes in diet are at least partly responsible for the trend. One factor that will have contributed to trends at young ages is the preventive effect of improved detection and treatment of familial polyposis coli.

### Cancer of the pancreas (Figure 8)

Mortality rates from cancer of the pancreas increased at all ages over 35 years until about 1970, but subsequently there was a decline in men and no clear trend in women. In incidence data for men (not shown) there was a similar pattern to that for mortality, but in women there was an appreciable increase since 1960 not seen for mortality.

Mortality data showed no great change between cohorts, but in the most recent cohorts, trends were irregular and confidence intervals wide. There was a small peak in men born in 1900–4 and women born in 1925–29. In incidence data by birth cohort (not shown) there was a similar pattern.

Since the prognosis of cancer of the pancreas is very poor [5-year survival remains under 5% (Black et al, 1993)], incidence and mortality trends for the tumour should be almost identical. For men this was approximately so, but for women there was a recent rise in recorded incidence not seen in the mortality data. This divergence raises the possibility that the rise in women is an artefact of improved registration, and examination of mortality/incidence ratios over time suggest that this may well be so: at ages 70–84 years, for instance, this ratio decreased from 138.5% in 1960–64 to 96.2% at the end of the 1980s, while 5-year survival remained under 5% (Black et al, 1993). In men there was also a decrease in the mortality/incidence ratio, but starting from a lower initial figure, which may indicate less initial under-registration.

The main known risk factor for cancer of the pancreas is smoking, but with a much lower relative risk than that for lung cancer. The slight peak of pancreatic cancer in men born in 1900–04 corresponds to the much larger peak of lung cancer in men in the same cohort, suggesting that smoking trends may have had an effect on the pancreatic cancer rates. In women, the peak cohort for lung cancer was that born in 1920–24, and for pancreatic cancer there was a peak for those born in 1925–29. The pancreas is a deep abdominal organ and diagnosis of pancreatic malignancy is not easy, so the trends, particularly at older ages, may have been affected by changes in completeness of diagnosis.

### Cancer of the larynx (Figure 9)

Mortality from cancer of the larynx in men showed a pattern reminiscent of that for oral and pharyngeal cancers. There was a large increase for men aged 35–49 years since 1970–74 (although not as large in proportional terms as that for oral and pharyngeal cancers), and at older ages a modest decline. In women aged 35–49 years rates declined to about a third of their original level, and at older ages there was no clear trend. Incidence trends, unexpectedly, were very different from this. In men there was an increase of about two-thirds at each age since 1960, whereas in women there was an increase of a half at ages 35–49 years, and a tripling of rates at ages 50 years and above.

In cohort mortality data (not shown), there were peaks in risk for men born in 1875–79 and those born in 1940–44, whereas in women there were peaks for those born before 1885 and those born in 1915–19. In cohort data for incidence (not shown) there was again a peak for men born in the 1940s, but for women there were peaks for those born in 1925–29 and perhaps 1940–44.

The increase in laryngeal cancer mortality in young men in recent years accords with the increases for oropharyngeal cancers and oesophageal cancers in this group, and seems likely to reflect smoking and drinking, as 80–90% of laryngeal cancers in Western countries can be attributed to these habits (Tomatis et al, 1990). As noted previously (see p. 4), the prevalence of smoking has decreased in young men in Scotland in recent years, and probably average smoking has decreased for several decades, so that the rise in laryngeal cancer is likely to reflect increased drinking, and/or an increased prevalence of individuals who both smoke and drink heavily, as the effect of alcohol plus smoking is approximately multiplicative.

The reason for the large divergence between incidence and mortality rates over time is not clear. Although it might in part reflect improved survival, the scale of difference is too large to be explained by the changes in treatment that have occurred, or by the changes in survival for the period for which data are available: between cases incident in 1968–72 and those incident in 1983–87, all-age 5-year survival increased from 48.6% to 53.0% (Black et al, 1993). It is possible that registration has improved greatly or that the diagnostic or coding boundaries for laryngeal cancers in incidence and mortality data have diverged over time; there is potential for this in the problematic discrimination between the larynx and adjacent sites coded to the oropharynx and hypopharynx (Muir, 1992).

### Cancer of the lung (Figures 10 and 11)

Mortality from lung cancer in men has declined at ages under 70 years since 1965–69, and at age 70–84 years since 1980–84. The greatest proportional decrease has been at the youngest ages: at

© Cancer Research Campaign 1998

**Table 2** Cutaneous malignant melanoma incidence rates[a] at ages 15–84 by anatomical site, Scotland, 1960–4 to 1985–90

| Site | Males | | 1985–90 rate as % of 1960–4 rate | Females | | 1985–90 rate as % of 1960–4 rate |
|---|---|---|---|---|---|---|
| | 1960–4 | 1985–90 | | 1960–4 | 1985–90 | |
| Head and neck | 0.9 | 2.2 | 243 | 0.7 | 2.0 | 274 |
| Upper limb | 0.3 | 1.5 | 600 | 0.3 | 2.2 | 721 |
| Trunk | 0.4 | 3.3 | 765 | 0.4 | 1.5 | 391 |
| Lower limb | 0.5 | 1.8 | 373 | 1.2 | 6.6 | 568 |
| Other and unspecified | 0.2 | 0.2 | 140 | 0.3 | 0.3 | 85 |
| Total, all sites | 2.2 | 9.1 | 405 | 2.9 | 12.7 | 434 |

[a]Rates per 100 000 population, age-standardized to the Scottish population of 1971.

age 15–34 years, rates are now less than a fifth of those in 1960–64. In women too there has been a decrease at the youngest ages (15–34 years), to about a third of the 1960–64 rate; at age 35–49 years changes have been small, but at older ages there have been large increases. As a result of the difference in trend between the sexes, the all-age mortality rate in women is now 41% of that in men, whereas 30 years ago it was only 13% of the contemporary male rate. For incidence (not shown), a similar pattern was present.

When analysed by cohort, there was a clear peak in mortality for men born in 1900–04 and women born in 1920–24 and then in each sex a decline that continued until at least the 1955–59 birth cohort. Incidence data were similar (not shown).

Longer term secular data for lung and pleural cancers combined (see Materials and methods) (Figure 11), which are effectively the trends for lung cancer as pleural cancer is comparatively uncommon, show low rates in each sex until the mid-1920s, and then in men a steep increase for 50 years to a peak in 1970–74, and in women a lesser increase, mainly since 1960, that has yet to reach its peak.

The rates of lung cancer in men in Scotland from the late 1950s to the 1970s were the highest national rates recorded in the world, and in women from the early 1950s to the early 1980s were the second highest, based on countries included in the compendium by Kurihara et al (1989). These rates reflect the high level of smoking in Scots born earlier in the century, and the trends are largely the result of changes in smoking habits (see p. 5) and in the tar yield of cigarettes, which has been decreasing in the UK since the Second World War (Wald et al, 1988). The recent decreases in rates are also, to a lesser extent, a reflection of decreases in air pollution in urban areas (Doll, 1990) and improvements in industrial hygiene. Domestic exposure to radon in air probably causes a few per cent of lung cancers, and as radon concentrations are increased by double glazing and other draught proofing, one may speculate that average radon concentrations in houses might have increased over time and provided a small counterweight to the decreases in smoking and the other risk factors described above. At older ages, at least, new diagnostic technologies – fibreoptic bronchoscopy, fine-needle biopsy, and computerized tomography – may have had some effect on rates. The overall effect, however, could have been either to increase or decrease rates: the new methods could have increased the number of lung cancers diagnosed, but also may have reduced the number of instances in which other lung pathologies were misdiagnosed as primary lung cancer (Gilliland and Samet, 1994).

### Cancer of the pleura (Figure 12)

Recorded mortality from cancer of the pleura has increased by more than 12 times in men and more than five times in women since 1958–59. There have been large increases in incidence too, especially in men aged 70–84 years (over 100 times).

In cohort mortality data the increase in men continued consistently up to the cohort born in 1940–44, whereas in women, based on small numbers, there was no increase beyond the cohort born in 1925–29. Incidence data by birth cohort (not shown) displayed a similar pattern.

Cancer of the pleura in Scotland is mainly the result of occupational exposure to asbestos, and the cancer trends reflect past trends in this exposure as well as, probably, improvements in completeness of diagnosis. The numbers of cases in the population can be expected to rise for about 20 years to come (Peto et al, 1995).

### Malignant melanoma of the skin (Figure 13)

Cutaneous melanoma mortality has more than doubled in each sex since 1953–54. In women the greatest increases have been at younger ages, whereas in men increases have been similar at each age-group. Incidence data show much greater increases than for mortality – over fourfold in each sex over the shorter period for which data are available.

There have been considerable differences in the rate of increase in melanoma incidence by anatomical site (Table 2): six- or sevenfold increases have occurred since 1960–64 for upper limb melanoma in each sex, trunk melanoma in men, and lower limb melanoma in women, whereas much smaller increases have occurred for other site–sex combinations.

In cohort data, the increase in all-site melanoma mortality in men took place in generations born up to 1930–34, and the increase in women in those born up to 1920–24, but subsequently there was no clear trend. In incidence data, there were large increases in women through to the most recent cohorts, whereas in men the increase was slight for those born after 1930–34.

The increasing incidence of melanoma in Scotland parallels similar increases in white populations worldwide, which have been occurring as far back as data have been available. Completeness and accuracy of melanoma registration data in Scotland is probably particularly high because the routine cancer registration system is

© Cancer Research Campaign 1998

supplemented by data from the Scottish Melanoma Group, an independent pathologist-based registration system (MacKie et al, 1992). The registered melanoma incidence rates in Scotland are appreciably above those for England and Wales, despite the higher latitude of Scotland; the difference is probably in part real, although better registration may also have contributed to it.

The mortality trends in Scotland are similar to those in other white populations, in which the rate of increase has been lower for mortality than incidence, and on a cohort basis rates have stabilized or started to decline in recent birth cohorts (NRPB, 1995). Epidemiological investigations around the world suggest that the increases in melanoma rates are largely real and are likely to be due primarily to changes in UV exposure behaviour, and particularly to increasing recreational sun exposure of untanned skin (NRPB, 1995). For the Scottish population, this is likely to be a mixture of more sunbathing and outdoor recreation while in Scotland, and more overseas holidays. There may also have been some effect from the rising use of artificial tanning lamps and beds.

The greater increase in incidence than mortality rates from melanoma is in part due to improved treatment and probably to earlier presentation, at a stage when treatment is more successful. It may also reflect an increasing tendency to biopsy borderline malignant lesions. Over the past 15 years, at least, the proportion of melanomas that are <1.5 mm thick at presentation has increased considerably (MacKie and Hole, 1996). All-age 5-year survival from melanoma in Scotland improved from 55.4% for cases incident in 1968–72 to 69.7% for those incident in 1983–87, and the improvement was particularly marked at the oldest ages – from 24.1% to 45.4% at age 75–84 years (Black et al, 1993). The divergence of incidence and mortality trends is probably not, however, due to changed pathological criteria for borderline malignant lesions: an international collaborative study of melanoma pathology, which included two centres in the UK, although none specifically in Scotland, concluded that changes in histological criteria for malignancy could not account for more than a small amount of the rising rates (van der Esch et al, 1991).

### Breast cancer in women (Figure 14)

Percentage changes in breast cancer mortality over the last 80 years have been small compared with those for most other cancers, but because breast cancer is so common the changes are of importance. At ages under 45, mortality increased to a peak in 1935–39, rose to another peak in 1975–84, and has since fallen. (Ages under 45 years are taken here to represent premenopausal ages. Ideally this analysis would divide the age groups at 50 not 45 years of age, but this is not possible with the age-categorization of data available from early in the century.) At age 45–84 years there was also a rise to a peak in 1935–39, and to another in 1985–89, and then a lower rate in 1990–93.

Examining trends since 1953 in more detail, there were increases of 20% at age 50–69 years and 10% at age 70–84 years, with a slight downturn at each age group in 1990–93, and at ages 35–49 years, a rise of about 30% and then a decrease.

The increases at post-menopausal ages have been larger for incidence than mortality. At age 35–49 years there was an increase in incidence up to 1980–84, followed by a small decrease, and at 15–34 years, a marked increase up to 1970–74, but then a decline.

In cohort mortality and incidence data a peak was reached for women born in 1930–34, with a decline since. Cohort data for incidence by single year of birth did not show any change in risk for those born or reaching the age of puberty during the depression of the early 1930s or the years of the Second World War.

Breast cancer is the most common cancer in women in Scotland, and the Scottish mortality rates are among the highest in the world (Kurihara et al, 1989). The increase in breast cancer mortality at older ages over the last 40 years is an important part of the increase in cancer mortality overall in older women, and is unlikely to be artefactual. This is not a site where the mortality data are likely to have been affected appreciably by changes in diagnostic technology or criteria. Breast cancer mortality data at older ages are particularly susceptible to artefacts of death certification and of coding selection of underlying cause of death (Grulich et al, 1995), but again this does not appear likely to explain most of the increase. Screening in recent years may have resulted in the detection of incident cases that would not otherwise have been known, or that would have been diagnosed later, but it is unlikely to have increased apparent mortality, and indeed in the long term should lead to a decrease in mortality. The reason for the increase in breast cancer for cohorts born up to the early 1930s is uncertain. Earlier age at menarche and increased height (or correlates of these), perhaps because of better nutrition in childhood, may in part be responsible, as may changes in childbearing patterns and in post-menopausal obesity. The effect of the increasing use of post-menopausal hormone replacement therapy is likely to have been slight (Ursin et al, 1994). There is evidence, mainly from international ecological correlations, that high fat consumption may be a risk factor for breast cancer; in the period for which national Scottish data are available, fat consumption increased from 95 g per head per day in 1953 (MAFF, 1955) to 114 g in 1969 (MAFF, 1971), and then decreased to 89 g in 1990 (MAFF, 1991). For the period before these National Food Survey data, aggregation of data from ad hoc studies suggests that the percentage of energy in the Scottish diet derived from fat increased considerably from the 1920s (25.0%) to the 1930s (34.9%), slightly decreased in the 1940s (33.5%), but then increased in the 1950s (38.6%) (Stephen and Sieber, 1994). These early dietary trends show a considerable parallel with the breast cancer trends, especially the interruption of the upward trend during the period of rationing during and after the Second World War.

Termination of pregnancy (therapeutic abortion) is another potential risk factor for breast cancer. Abortion became legal in Scotland in April 1968, since when abortion rates have risen from 3.5 per 1000 women aged 15–44 in 1969 (ISD, 1977), to 7.3 in 1980 (ISD, 1982), and 9.8 in 1990 (ISD, 1991).

The decrease in incidence of breast cancer in the most recent birth cohorts in Scotland corresponds with a decrease seen also in England and Wales (dos Santos Silva and Swerdlow, 1995). Data on age at first birth in the Scottish population are available only since 1976, and show an increase from an average of 23.1 years in 1976 to 25.7 years in 1995 (ISD, 1997) – a pattern that might be expected to contribute to an increase, not a decrease, in breast cancer incidence. The mean fertility by age 45 years of women in Scotland increased from 2.51 liveborn children per woman for women born in 1931 to 2.63 for those born in 1934, but then decreased steadily to 2.03 for women born in 1951 (RG for Scotland, 1996); more recent cohorts have not yet reached age 45 years, but up to the age they have currently attained their fertility rates have continued to decline. Like the age at first birth trends, the fertility trends are those that would accord with a rising, not falling, incidence of breast cancer.

The decrease in breast cancer incidence has coincided with the introduction and widespread use of oral contraceptives, which would not accord with an aetiological effect of oral contraceptives.

 © Cancer Research Campaign 1998

It is not due to the national screening programme, which was only for women aged 50 years and above. The quality of breast cancer registration data appears to have been high throughout: the percentage of cases under age 65 years histologically verified has increased from 90% in 1960–64 to 95% in 1985–89, and the percentage at these ages registered solely from death certificates has remained under 2%. The recent downturn in breast cancer mortality at younger ages is due partly to the downturn in incidence, but at older ages incidence has not decreased. At least in part the decrease in mortality is the result of better treatment by tamoxifen and other new effective treatments (Stewart, 1992; Early Breast Cancer Trialists' Collaborative Group, 1992). All-age 5-year survival from breast cancer in Scotland increased from 49.5% for cases incident in 1968–72 to 56.3% for those incident in 1983–87, with similar rates of improvement at each age-group (Black et al, 1993). The national breast cancer screening programme in Scotland was introduced too recently to account fully for the decrease in mortality, and for incidence it might well initially have given an artefactual increase (Ursin et al, 1994). The programme in Scotland attempts to screen by mammography all women aged 50–64 years every 3 years, and to screen women older than this 3 yearly on demand. The first centres opened in 1988, and national coverage was obtained in 1991 (ISD, 1993); in 1991–92, the first year for which reliable data are available, 91 028 women were screened, and 437 invasive malignancies detected (ISD, 1993), compared with around 2000 breast cancers per year registered in women aged 50–84 years in the late 1980s.

### Cancer of the cervix uteri (Figure 15)

Trends in mortality from cervical cancer before 1970 were uneven, but since then there has been a clear decrease, except that at ages under 35 years rates have been erratic, based on small numbers. Incidence data show a similar pattern for ages over 35 years, but at ages under 35 years there has been a dramatic increase since 1960, with the rate more than tripling.

Cohorts born in 1910–24 had high mortality, there was then a steep decrease to a minimum for the cohort born in 1930–34, and an increase to the 1955–59 birth cohort, the most recent for which there was a substantial number of deaths. Incidence data by cohort were similar, but in addition displayed a clear decrease in the cohorts born in the 1960s.

Cervical cancer trends are potentially open to artefact if the proportion of uterine malignancies whose exact site is not specified is high, especially if this proportion changes. For incidence, the proportion was low (5.8%) and did not change substantially over time; for mortality, the proportion has been greater (19.6%), and has decreased from 43.2% in 1953–54 to 9.4% in 1990–93. At all ages combined, judging from English and Welsh experience (Swerdlow, 1989), most of the unspecified tumours are likely to have been of the corpus uteri, not the cervix, so that although there was probably an increase in recorded mortality due to greater precision of death certificate diagnoses, this effect is unlikely to have been large. At young ages, however, the great majority of uterine cancers are cervical, so that there is a greater chance that uterine cancers not further specified were in fact cervical: for instance at ages 15–34 years in 1990–93, 97% of uterine cancer deaths were stated to be cervical, and at ages 35–49 years, 91% were stated as cervical, so at these ages uterine deaths not further specified may well have included a substantial proportion of cervical cancers. The unspecified deaths diminished from 17% to 3% of all uterine cancer deaths at age 15–34 years, and 31% to 4% at age 35–49 years, from 1953–54 to 1990–93, and this may well have had an appreciable upward effect on the cervical cancer mortality trends.

For incidence, however, the effect will have been slight as few uterine cancers at young ages were of unspecified subsite – under 1% at age 15–34 years, and under 2.5% at age 35–49 years. The quality of cervical cancer registration data also appears to have been high: well over 95% of cases in 1960–89 at ages under 65 years and around 85% of cases at ages 65–84 years had histological verification, and under 1% of cases under age 65 years and around 2–3% of cases at ages 65–84 years were registered from a death certificate only.

Cervical cancer rates may also be artefactually influenced by diagnostic and coding boundaries between cervical malignancy and cervical intraepithelial neoplasia (cancer in situ and dysplasia), especially as screening will reveal many cases of less severe disease. We have no data on whether these boundaries have altered, but it seems unlikely that this could explain the great variation in trend by age in recent years.

The decreases in cervical cancer at older ages in Scotland accord with decreases in many Western countries, probably mainly because of changes in sexual behaviour, although there may have been some effect also of declining parity (on which data are not available for cohorts born before 1931 – see p. 8) and possibly of better genital hygiene. Rates in recent years are likely also to have been reduced by screening. The cervical screening programme started in certain parts of Scotland in the early 1960s (Duguid et al, 1985; MacGregor et al, 1985), but was uneven in coverage until a computerized call–recall system was introduced in the late 1980s. The smear rate, per 1000 women aged 15 and above, increased from 74 in 1967 (ISD, 1982) to 133 in 1980 (ISD, 1982) and 261 in 1990 (ISD, 1991). Rates may also have been diminished by a rising frequency of hysterectomy: the age-standardized hysterectomy rate (European Standard Population) increased from 150.7 per 100 000 per year in 1961–65 to 241.2 in 1976–80 and 288.5 in 1991–95 (ISD, unpublished), which will have reduced the numbers of uteruses at risk of cancer. Smoking has been found associated with cervical cancer risk, although whether causally is uncertain; cervical cancer trends resembled those for lung cancer, as a marker of smoking, in that there was a peak of incidence in women born around 1920, but the rise in recent cohorts for cervical cancer was not present for cancer of the lung.

The increase in cervical cancer incidence in Scottish women born from the Second World War onwards, who reached sexual maturity during the period when oral contraceptive use became widespread, has been seen also elsewhere in the British Isles, and in Australia, New Zealand and eastern Europe (Beral et al, 1994). Its presence in mortality as well as incidence data suggests that it is not due to changes in diagnostic criteria for borderline malignant lesions or to case finding from screening. The increase is likely to be due to changed sexual behaviour. Supporting this is the fact that it occurred at the time of a large upsurge in venereal disease – there were 1768 new cases in women presenting to clinics in Scotland in 1950, and 1888 in 1960, but this increased to 4251 in 1970 and 7314 in 1973 (ISD, 1977). An element of the increase might also have been a direct effect of oral contraceptives on risk, although it is uncertain whether this association is causal.

The steep decrease in cervical cancer incidence in cohorts born in the 1960s, based on moderate numbers of cases, has not to our knowledge been reported for other countries. It might indicate the

© Cancer Research Campaign 1998

effect of screening (although this is intended to be primarily for older women) and/or an effect of AIDS on sexual behaviour (including barrier contraceptive use). Recent data on sexually transmitted disease clinic attendances give some support to the latter interpretation. Peak attendance rates in women under age 35 years were reached in 1985–87 (ISD, 1989), and then decreased by 13–16% (depending on age) by 1990 (ISD, 1991).

### Cancer of the ovary (Figure 16)

Ovarian cancer mortality has increased at older ages over the past 40 years, but at younger ages there was little change up to the 1970s, and then a marked decline, especially at ages under 35 years. The increase at older ages was present, and indeed slightly more marked, in incidence data. At young ages there were slight increases in incidence.

In cohort data an increase in mortality was seen in generations of women born up to 1915–19, with a decline thereafter. In incidence data (not shown), there was an increase from cohorts born late in the last century until the 1925–29 cohort, but subsequently no clear trend.

The decrease in mortality from ovarian cancer at younger ages while incidence has not diminished, reflects the introduction of effective chemotherapy in the 1970s: at age 35–44 years, 5-year survival improved from 35.1% for cases incident in 1968–72 to 56.2% for those incident in 1983–87, and there were lesser improvements at age 45–54 years and 55–64 years (Black et al, 1993). The lack of a similar divergence between incidence and mortality trends at older ages, however, implies that the effect of chemotherapy has been small, on a population basis, at these ages. Again this accords with the available survival data: from cases incident in 1968–72 to those incident in 1983–87, there was no material improvement in survival at age 65–84 years (Black et al, 1993).

Trends in ovarian cancer incidence (other than to the extent they are artefacts of diagnosis or registration) are likely to reflect mainly changes in parity, age at menarche and in recent cohorts, oral contraceptive use. Parity has been decreasing, at least for cohorts born since the mid-1930s (see p. 8). The rates will also have been somewhat affected by hysterectomy and oophorectomy rates as these affect the number of ovaries at risk. The rate of hysterectomy, which often includes removal of the ovaries, has increased greatly since 1961–65 (see p. 9), and the rate of oophorectomy without hysterectomy increased from 62.6 per 100 000 (standardized to the European Standard Population) in 1961–65 to 90.5 in 1981–85, and then decreased to 72.3 in 1991–95.

In England and Wales and in Sweden, decreases in ovarian cancer incidence have been seen in recent cohorts, probably consequent on increased use of oral contraceptives (Adami et al, 1990; Dos Santos Silva and Swerdlow, 1995). There is no clear evidence that this has yet occurred in Scotland.

### Cancer of the prostate (Figure 17)

Mortality from cancer of the prostate at age 50 years and above changed little until the early 1980s, but has since increased appreciably. At younger ages, deaths from this malignancy are rare and rates have been erratic. Incidence rates have risen more steeply than mortality.

Changes in mortality from prostatic cancer in cohort data (not shown) have been slight, except for an apparent increase, based on small numbers, in the most recent birth cohorts. In incidence data by cohort (not shown), rates rose for the cohorts born up to 1925–29, and were erratic thereafter.

The large secular increase in recorded incidence of prostatic cancer has been seen in many countries and is likely mainly to reflect increased detection through the increasing use of transurethral resection (TURP) to treat benign prostatic hypertrophy (Potosky et al, 1990). This increased detection during life might also have had some effect on recorded mortality rates. The proportion of registered cases for which histological verification was available increased greatly, from 55% in 1960–64 to 91% in 1985–89 at age 35–64 years, and from 51% to 83% at age 65–84 years. The introduction of prostate-specific antigen screening in the last few years of the study period may also have had some effect. Autopsy can reveal 'latent' prostatic malignancies that would not otherwise be detected. For the period for which data are available, however, autopsy rates have decreased slightly – from 16.2% of deaths in 1965, to 15.9% in 1980 and 14.5% in 1994. It may also be the case that changes in certification practice account for part of the rise in recorded underlying cause mortality from this cancer, as appears to have occurred in England and Wales (Grulich et al, 1995). Nevertheless, it seems likely that mortality rates will have been less affected than incidence rates by artefacts of incidental diagnosis of latent tumours, and changes in certification seem unlikely to explain the scale of the mortality rise, so it may well be that the increase is at least in part real, although too little is known about prostatic cancer aetiology to assess the factors that might be responsible.

The divergence between incidence and mortality trends is what would be expected if TURP has led to greater incidental diagnosis of relatively good prognosis tumours. Surprisingly, however, it does not accord with survival data, which one might then expect to show apparent improvements, as more-benign tumours were added to the 'incident' cases. The published survival data show little change: all-age 5-year survival was 27.8% for cases incident in 1968–72 and 30.5% for those incident in 1983–87 (Black et al, 1993).

### Cancer of the testis (Figure 18)

Data on mortality from cancer of the testis in Scotland are available back to 1931. At age 50 years and above, rates were erratic until the 1950s but have since decreased by a half. At ages under 50 years, rates more than tripled from 1931–35 to 1976–80 but subsequently decreased steeply, by about two-thirds. Analyses for finer age groups than in Figure 18 showed similar patterns. Incidence rates of cancer of the testis since 1960 have changed little at older ages, but more than doubled at ages under 50 years.

In cohort mortality data (not shown), there have been generally downward trends for men aged 50 years and above, whereas at ages under 50 years a peak was reached for the cohort born in 1935–39, with a large decline subsequently. Cohort trends in incidence for men aged 50 years and above (not shown) were uneven, based on small numbers; for men under 50 years there was an increase up to the cohort born in 1960–64, but no further rise for those born subsequently. Cohort data by single year of birth (not shown) gave no indication of an altered risk for men born during the Second World War or the depression of the 1930s.

Increasing incidence of testicular cancer at younger ages has been seen in white populations around the world for as long as reliable data have been available (Swerdlow, 1997). There is no reason to believe that diagnostic factors could have had a material effect, or that registration improvements could account for more than a minority of the increase, at most. The reason for

© Cancer Research Campaign 1998

the rising rate is not known. Cryptorchidism accounts for too small a proportion of testicular cancers for changes in its frequency to explain the increase in testicular cancer. Increasing in utero exposure to environmental oestrogens, affecting development of the testis (Sharpe and Skakkebaek, 1993), or decreasing age at puberty and increasing sedentariness and lack of exercise (Forman et al, 1994), have been recently hypothesized as causes, but there is insufficient knowledge about their aetiological roles or data on their trends to assess if they can account for the rising risk. There was no sign in the Scottish data of the halt in the rising cohort trend for men born during and soon before the Second World War that has been observed in Denmark, Norway and Sweden (Bergström et al, 1996), but the effect of the War on nutrition was probably less severe in Scotland (which was not occupied) than in occupied Denmark and Norway, at least.

The decreasing mortality from testicular cancer at older ages over many decades, and the decrease in mortality at young ages since the 1970s, are trends that have been found elsewhere. The decrease in mortality in recent years relates to improved survival from the introduction of effective chemotherapy. Comparing cases incident in 1968–72 with those incident in 1983–87, all-age survival improved from 60.7% to 86.8% (Black et al, 1993), and there were especially large improvements in young adults – from 42.6% to 89.5% at age 15–24 years, and from 65.0% to 91.5% at age 25–34 years. There were probably also substantial improvements in survival in the years before 1968 as a consequence of the introduction of intensive radiotherapy for seminomas.

### Cancer of the bladder (Figure 19)

We have included in the analyses of bladder cancer, cancers of the urethra and certain other non-renal urinary tumours, to improve the degree to which the same group of cancers can be identified ('bridged') in different ICD revisions. The analytic category is effectively bladder cancer, however, as these contribute over 98% of cancers within the codes analysed.

Mortality from cancer of the bladder at ages under 70 years showed no consistent trend (the apparent early decrease for women aged 35–49 years is based on few deaths). At age 70–84 years, however, rates in men have almost doubled and in women have increased by a half since 1953–54. Increases in incidence have been seen at all ages older than 35 years, with the largest increase at the oldest ages.

Mortality changed little between cohorts born late in the last century and those born in 1930–34, but for subsequent cohorts there was a small decline (not shown). In incidence data, there were increases up to cohorts born in the 1920s, and a generally downward trend for cohorts born subsequently.

The main known risk factor for bladder cancer is smoking, but in men neither the incidence nor the mortality trends paralleled those for lung cancer, for which cohort-based data showed a peak for men born in 1900–04. The cohort data in women, for incidence at least, showed a greater resemblance to the pattern for lung cancer, with a peak in those born in 1920–24. Trends in occupational chemical exposures and phenacetin use might affect bladder cancer trends (Silverman et al, 1996) as might, to a lesser extent, trends in pelvic radiotherapy and in chemotherapy, notably with cyclophosphamide, but there are no data on changes in these exposures over time in Scotland, except that manufacture and use of certain aromatic amines ceased at several large companies in Britain several decades ago (Swerdlow, 1990).

The divergence between incidence and mortality trends for bladder cancer accords with improvements in recorded survival: from cases incident in 1968–72 to those incident in 1983–87, all-age 5-year survival increased from 40.1% to 48.4% (Black et al, 1993). This improvement could indicate better results of treatment, or the inclusion of greater numbers of non-aggressive tumours in the incidence data, which could artefactually have increased incidence rates. This might have occurred if clinicopathological terminology or criteria for papillomas of the bladder (Saxén, 1982) or registry interpretation or coding of invasiveness of bladder tumours (Lynch et al, 1991) had changed over time; we have no data on whether this occurred, and the data available to us were solely for tumours coded as malignant.

### Cancer of the eye (Figure 20)

At age 15 years and above, ocular cancers are largely melanomas, and at younger ages ocular melanomas rarely occur, so ocular cancer trends at 15 years and above give a good proxy for trends in ocular melanoma (Hakulinen et al, 1978). Ocular cancer mortality at age 15–84 years in men showed no clear trend. In women, there was an increase from 1953–54 to 1970–74, and perhaps a subsequent decrease. Incidence data by contrast showed substantial increases, especially in recent years, with rates in each sex in 1985–90, 60% greater than those in 1960–64. We have not shown age-specific trends in ocular cancer because of small numbers. The mortality trends did not vary clearly by age, but incidence rates tended to increase most steeply at the oldest ages.

The apparent rise in incidence of melanoma of the eye during the 1980s may be an artefact as developments in the ophthalmic oncology service in Scotland around this time attracted many referrals of patients from outside Scotland, some of whom may inadvertently have been registered as if Scottish residents.

Whereas the aetiology of cutaneous melanoma is known to be related closely to UV exposure, the relation of ocular melanoma risk to UV is far less certain (NRPB, 1995). It is notable in this respect that if there has been an increase in eye cancer incidence in Scotland over the past 30 years (and it is uncertain how much of the apparent increase is due to the above artefact or improved registration), it has clearly been much less than the increases that have occurred in cutaneous melanoma rates. By implication, the increase in recreational and vacational intermittent sun exposures that is believed to be the main cause of the rise in cutaneous melanoma has had far less, if any, effect on ocular melanoma incidence trends.

The difference between the incidence and mortality trends for ocular cancer might have occurred because of artefact in the incidence data (see above) or improved cancer registration, or there may have been truly increasing incidence counterbalanced by improved survival as a reason for the approximately unchanged mortality. No survival data are available for cancers of the eye in Scotland.

Ocular melanomas are sometimes stated on death certificates simply as 'melanoma', and if the correct site is not ascertained they will then be coded under the ICD rules to a category within cutaneous melanoma, rather than to ocular malignancy. The large increase in rates of cancer of unspecified site in Scotland over the last 40 years suggests that specificity of death certification has deteriorated, so that specification of site for ocular melanomas may also have become worse. The effect is likely to have been small (Swerdlow, 1989), however, and is not likely to account for most of the observed divergence between ocular melanoma incidence and mortality.

© Cancer Research Campaign 1998

### Cancer of the nervous system (Figure 21)

Certified mortality from nervous system cancer increased several-fold at age 70–84 years over the study period, especially in men, but there were far smaller increases at age 50–69 years, and small decreases in younger adults. In boys but not girls there has been a decrease in recent years. The differences between age groups in nervous system cancer trends have been so large as to change the cross-sectional age distribution of mortality from this cancer in Scotland – in the 1950s a peak was reached at age 50–69 years, with a decrease at older ages, whereas now rates rise progressively with increasing age in a manner similar to that for most cancers.

In incidence data too (not shown) there have been large secular increases in nervous system cancer rates at older ages, although less pronounced than those for mortality. There has also been a moderate increase at younger adult ages for men but not women, and increases for children of each sex. As a result of the larger increases for mortality than incidence at age 70–84 years, recorded mortality rates at these ages are now, surprisingly, appreciably greater than recorded incidence.

In birth cohort data, a large increase in mortality was seen up to cohorts born around 1915–19, with a slight decrease for those born thereafter. The age distribution within cohorts has altered, from a peak around age 65 years to a pattern of rising rates with increasing age. In incidence data (not shown), there was also an increase for cohorts born up to the early years of the century, but in addition an increase for males born since 1960–64 and females born since 1965–69.

The above analyses do not include benign and unspecified tumours of the nervous system because incidence data on these were not available and we wished to make the incidence and mortality analyses comparable. It is desirable to consider benign and unspecified as well as malignant tumours when examining nervous system cancer trends, however, because they frequently cannot be distinguished in patients in whom the diagnosis is made without biopsy. When benign and unspecified tumours were included in the mortality analyses, the trends were similar to those shown, unsurprisingly as they constituted only 15% of deaths (under age 85), but with a tendency for the percentage increase at older ages to be greater when these tumours were added. Coding of brain tumours as malignant in mortality data is considerably dependent on the precision of information furnished by certifiers (Swerdlow, 1989) (and the same is presumably true for registration data), so that the greater increase for all brain tumours than for those specified as malignant might be a consequence of changed certification practice.

The large apparent increases in incidence and mortality from nervous system cancers at older ages in Scotland have been seen also in many other countries (Ahlbom, 1990; Muir et al, 1994). They have probably been caused, at least in part, by better diagnosis, for instance through the introduction of computed tomography scans and magnetic resonance imaging; judging from North American data, however, these latter modalities may only account for a minority of the increase (Desmeules et al, 1992). If biopsy rates have decreased, then rising rates could be due to some extent to more secondary malignancies in the brain being misclassified as primary brain tumours – the proportion of registered nervous system cancers that were histologically verified has decreased from just over 80% in each sex in 1960–64 to 71% in males and 66% in females in 1990–94. It is implausible that the large change between cohorts in the age distribution of mortality can have been other than artefactual. Whether in addition to this artefactual increase there has also been a real increase in incidence is uncertain.

The larger increases in recorded mortality than incidence rates at older ages is paralleled to some extent by changes in published survival from nervous system cancers, which at age 45 years and above has deteriorated over the period (Black et al, 1993) (whereas at younger ages there has been a considerable improvement). This apparent deterioration in survival, and the lesser increase over time for incidence than mortality, could have occurred if better diagnosis or registry data collection had reduced the number of benign brain tumours or other brain lesions of good prognosis, categorized as brain malignancies. Autopsy can uncover considerable numbers of brain tumours at older ages undiagnosed during life (Muir et al, 1994), and thus decreasing autopsy rates could reduce the number of diagnosed non-fatal tumours; in the period for which data are available, there was a slight decrease in the autopsy rate (see p. 10).

### Hodgkin's disease (Figure 22)

Mortality from Hodgkin's disease increased until 1970 and subsequently declined by at least a half at each age group in each sex, except that for men aged 70–84 years the decline was less pronounced. Incidence rates at age 50 years and above have decreased by less than the decrease in mortality or (in men aged 70–84 years) not changed appreciably. At younger ages, incidence trends have been inconsistent.

In cohort mortality data (not shown) there were decreases since the cohorts born in 1885–89, but in incidence rates by cohort (not shown) there was little change.

The decrease in mortality from Hodgkin's disease since 1970, while incidence has decreased less or not at all, depending on age, is the result of the great improvement in prognosis brought about by intensive chemotherapy and radiotherapy. Judging from published time trends in survival, however, this only provides an explanation for the differing incidence and mortality trends at younger ages: at ages under 65 years there were considerable improvements in survival for cases incident in 1983–87 compared with those incident in 1968–72 (Black et al, 1993), whereas at age 65 years and above survival barely changed.

The decrease in incidence rates at older ages may in part reflect changed pathological classification of certain histological subtypes of Hodgkin's disease as non-Hodgkin's lymphoma (Banks, 1992). Recorded rates would also be affected if the specificity of reporting (or of registry data extraction) of histology of lymphomas has altered over time as lymphomas not further specified are categorized in the ICD under the rubric for non-Hodgkin's lymphoma, not Hodgkin's disease. The decreasing incidence and mortality at older ages could also to some extent reflect decreasing autopsy rates (see p. 10) as there is evidence that an appreciable proportion of cases of Hodgkin's disease at older, but not younger, ages are diagnosed only at post-mortem (Hasle and Mellemgaard, 1993).

### Non-Hodgkin's lymphoma (NHL) (Figure 23)

Mortality from NHL at ages 50 years and above has more than doubled since 1953–54. At younger ages, in contrast, there have been slight or no increases. Incidence has increased at all ages, and the upward trend has been steeper than that for mortality, except at age 70–84 years in women.

© Cancer Research Campaign 1998

In incidence data by birth cohort (and mortality data, not shown), rates in each sex increased steeply up to the cohort born in 1915–19, changed little until the birth cohorts of the 1950s, and then decreased.

The increase in NHL rates in Scotland accords with an increasing trend in many other countries around the world (Devesa et al, 1992), the aetiology of which remains largely unexplained. A small element arises from lymphomas due to immunosuppression for transplantation and in recent years from AIDS-associated lymphomas. These are relatively rare causes, however, especially at older ages. A small proportion of cases are of other known causation – adult T-cell leukaemias–lymphomas due to human T-lymphotropic virus type 1 (HTLV-1) (which might have been coded to leukaemia or NHL in Scotland during the period), and lymphomas in persons with genetic immunodeficiency or immune-related diseases such as coeliac disease; these causes are also uncommon, however, and cannot account for the large rise in rates. Other factors have been suggested as possible causes of the increase – exposure to pesticides and phenoxyherbicides, and to ultraviolet radiation or other factors that might alter immune status – but it is not yet clear whether they are aetiological.

Certain artefacts might explain some of the increase, but again not most of it. There may have been a transfer to NHL in recent years of some lymphomas that previously would have been categorized as Hodgkin's disease (Banks, 1992). Small changes in lymphoma rates could also have arisen from changes in classification between NHL and certain leukaemias, and there has been recategorization as malignant of certain (relatively uncommon) lymphoid lesions that were previously considered benign (Banks, 1992). Better specification or coding of lymphomas of sites other than lymph nodes (e.g. of the stomach or testis) might also have had a minor effect. Under ICD rules such tumours should have been coded to 'lymphoma' if the histology was stated, but if the histology was not specified on the death certificate or cancer registration, or the ICD rules were not followed correctly, then they could have been coded to the site at which they occurred. Judging from the results of a pathology review of cases in part of England over a 20-year period (Barnes et al, 1986), however, neither changes in pathological criteria nor changes in completeness of registration or coding can explain the increased rates.

It is possible that some of the increase might have occurred through improved diagnosis of cancers that were previously misdiagnosed as primaries of other sites or were categorized as malignancies of poorly defined or unknown site, or that remained undiagnosed. However, the sharply increasing rates of cancers of unknown primary site, one of the most obvious sources of tumours that might prove to be NHL with more precise diagnosis, are scarcely supportive of this. On the other hand, the increase in rates at older but not younger ages, for mortality in each sex and less convincingly for incidence in men, is a pattern that might reflect improved diagnosis (or a cohort effect from an aetiological agent acting less in recent years).

Given the lack of plausible artefactual explanations for the increase in NHL and the large scale of the increase, it seems likely that at least part of it has been real, due to as yet uncertain causes.

The divergence between trends in incidence and mortality from NHL, especially at younger ages, reflects primarily the impact of the introduction of effective chemotherapy and radiotherapy, which can generally be used more aggressively and successfully at younger ages. Five-year survival at age 35–44 years improved from 38.9% for cases incident in 1968–72 to 60.5% for those incident in 1983–87, and there were similar increases at other ages under 45 years, whereas at age 75–84 years the improvement was from 9.3% to 15.1% (Black et al, 1993). Some of the divergence between incidence and mortality trends might also be attributable to improved completeness of cancer registration.

### Multiple myeloma (Figure 24)

Myeloma mortality has increased sixfold in men and fourfold in women at age 70–84 years since the early 1950s, and has increased twofold or less at age 50–69 years. Few myeloma deaths occurred at younger ages than this. Trends in incidence (not shown) at age 70–84 years have been similar to those for mortality, but at age 50–69 years there has been a greater increase than that seen for mortality, and at 35–49 years there has also been an increase.

In cohort mortality data, there were large increases until those born early in this century, and less marked decreases subsequently. The shape of the age curve did not change appreciably between cohorts. In incidence data (not shown) there was a similar pattern to that for mortality, except that the decrease since the peak cohorts was less pronounced in men and not present in women.

The increases in myeloma rates in Scotland, greatest at older ages, are similar to those seen in many developed countries (Cuzick, 1994), and have no known aetiological explanation. Definitive diagnosis of myeloma requires relatively sophisticated tests. Increases internationally have been greatest where rates were initially low, and at ages (the elderly) when there was scope for better ascertainment (Cuzick, 1994), and rates have increased modestly or not at all in places where there was originally especially good ascertainment (Linos et al, 1981; Turesson et al, 1984). It therefore seems likely that although there might be an element of real increase, much or all of the rise in rates internationally may be an artefact of improved diagnosis. There appears to be no reason why the same interpretation should not apply to the Scottish results.

The differences in trend between incidence and mortality data may well be artefactual: recorded survival from myeloma has scarcely changed over the period (5-year all-age survival was 13.2% for cases incident in 1968–72 and 15.4% for those incident in 1983–87 (Black et al, 1993)).

The cohort analyses imply that if there is a real element to the rise in recorded myeloma rates, the aetiological factors concerned have long ceased to increase – the upward trend in myeloma stopped or even reversed for cohorts born after the early part of this century.

### Leukaemia (Figures 25 and 26)

Leukaemia mortality has increased at age 70–84 years except in recent years, at age 50–69 years in men and 35–69 years in women it has remained almost unchanged, and at younger ages it has decreased considerably. In incidence data there were again large increases at older ages, but also moderate increases at most younger ages.

In cohort mortality data (not shown) there were two peaks in each sex, the first for cohorts born at the turn of the century, and the second for those born in the 1950s. In incidence data by birth cohort there were less convincing peaks for cohorts born early in this century and in 1945–49, but, in addition, especially in men, there was an increase in cohorts born since 1970. Because this latter increase was composed of young age groups (necessarily, since based on recent years of birth) we reanalysed cohort incidence data only for age 0–24 years (not shown); in these analyses, increases occurred for males born since 1965–69 and females born since 1960–64.

Examining the secular data by histological type of leukaemia and single year of age (Figure 26), the increase in leukaemia at young ages occurred mainly for lymphoid leukaemia, and although it was present throughout childhood, it was most evident numerically at the peak incidence in young children around age 3–4 years.

The increase in leukaemia incidence and mortality at age 70–84 years in the early part of the study period, but ceasing more recently, is a pattern seen in several other Western countries (Kinlen, 1994). It may in part or entirely be a result of improved diagnosis and registration, although a real increase in incidence cannot be discounted. The known causes of leukaemia in adults – ionizing radiation, alkylating chemotherapy, benzene and probably chloramphenicol – account for relatively few cases, and could not explain the increase. Cigarette smoking may be a cause of leukaemia, but the leukaemia trends did not resemble those for lung cancer as a proxy for smoking. The large decline in mortality at younger ages is the consequence of improved treatment by chemotherapy and bone marrow transplantation. Although leukaemia survival has improved at all ages over the last 25 years, the greatest improvements have been at the youngest ages: at age 0–4 years, 5-year survival increased from 21.0% for cases incident in 1968–72 to 74.3% for cases incident in 1983–87, compared with a rise from 12.5% to 22.5% at age 35–44 years, and from 12.0% to 16.9% at age 65–74 years (Black et al, 1993).

The increase in childhood leukaemia incidence is of concern. Registration completeness for this tumour has been particularly good (Glass et al, 1987), so the rise does not appear to be attributable to better registration. In other countries, data have provided limited evidence for an increase in acute lymphocytic leukaemia (ALL), at least at young childhood ages, which, 'if real, seems to have occurred at different times in different countries and to be small' (Draper et al, 1994). The known causes of childhood leukaemia – Down's syndrome and certain other rarer chromosome abnormalities, and ionizing radiation, probably including intrauterine irradiation – would not explain the increase. The average annual radiation dose in the UK from weapons fallout increased from the late 1940s, when the first nuclear weapon explosion in the atmosphere took place, to the mid-1960s, and decreased thereafter when the test ban treaty was implemented (Hughes et al, 1989). The levels increased slightly again after the 1986 Chernobyl accident. As noted previously for Scottish data up to 1984 (Darby and Doll, 1987), there has been no relationship between leukaemia trends and levels of fallout. It has been hypothesized that population mixing leading to increased infection rates may be a cause of childhood leukaemia, and raised rates have been found in certain parts of Scotland where there has been a high level of population mixing associated with the North Sea oil industry (Kinlen et al, 1993). It is not clear, however, whether population mixing has increased in Scotland overall, as a potential explanation for national trends in childhood leukaemia.

### Cancer of unspecified primary site (Figure 27)

Mortality ascribed to cancer of unspecified site has increased greatly at all ages, but especially at older ages since 1970–74. In 1953–54, unspecified malignancies constituted 0.7% of all cancer deaths (excluding non-melanoma skin cancer) at age 15–84 years, but by 1990–93 this had risen to 7.5%. Incidence rates for cancer of unspecified site have also increased at older ages, but not as greatly as mortality. Unspecified site malignancies constituted 3.4% of all cancers other than non-melanoma skin cancer registered incident at age 15–84 years in 1960–64 and 5.1% in the late 1980s.

One artefact affecting cancer of unspecified site trends has already been discussed (see p. 3): a temporary increase in registrations in 1966–67. As well as this artefact, there has been a background continuing increase in rates of cancer of unspecified site. This might reflect a tendency toward poorer specification of site of cancers on cancer registrations and death certificates, or toward less intensive investigation of primary cancer site in persons with terminal cancer of unknown origin. Autopsy rates have decreased in Scotland since 1965 (see p. 10), but by a far smaller proportion than the increase in cancer of unspecified site. Changes in the procedure for 'medical enquiry' (see p. 3) to certifiers when the cause described on a death certificate is imprecise could alter rates for imprecise cause categories, especially cancer of unspecified site, but no substantial changes to the procedure have occurred, at least since the early 1970s. Although it is possible that some of the increase in cancers of unspecified site might have been due to an increase in the incidence of cancers that metastasize early and aggressively, the rapidity of the change in rates, especially for mortality, suggests that it must mainly have been an artefact. The much larger rate of unspecified site cancer mortality than incidence in recent years also argues that much of the mortality increase is artefactual.

### CONCLUSIONS

Cancer mortality in Scotland has been high, with the greatest rates of lung cancer in the world and almost the greatest breast cancer rates. As Figure 28 shows, these two cancers dominate Scottish cancer mortality and incidence. It is encouraging, however, that for lung cancer in men and breast cancer in women the secular trend has now turned downward, and for lung cancer in women, although rates are continuing to rise, there is a decreasing trend on a cohort basis, and therefore secular rates should turn down in the longer term.

At young ages the decreasing trends in mortality from testicular and ovarian cancers, Hodgkin's disease and leukaemia are an encouraging result of advances in treatment. The increases in incidence of several cancers – upper aerodigestive tract cancers in men, melanoma, cervical cancer, testicular cancer, NHL and childhood leukaemia – however are of concern, although the recent cessation of rise in melanoma mortality and downturn in NHL and cervical cancer incidence, on a cohort basis, give room for optimism on future secular trends of these. The increasing rates emphasize the need for preventive actions for known risk factors, such as alcohol and tobacco use in relation to upper aerodigestive tract cancers, and for aetiological study especially of those cancers that are increasing for unknown reasons.

### ACKNOWLEDGEMENTS

We thank the Cancer Research Campaign and Medical Research Council for funding. Dr Calum Muir gave wise advice at the outset of this project, but sadly did not live to its conclusion. We are appreciative of the work of cancer registration staff throughout Scotland who have worked over many years to produce the high-quality cancer registration data that make this Supplement possible, and also of the mortality coding staff at the General Register Office. We thank Ms Linda Sharp for extraction of cancer

© Cancer Research Campaign 1998

incidence data, and colleagues in ISD Scotland for provision of unpublished statistics. We are grateful to the Carson-Clarke Gallery of Edinburgh for the copy of the map on the front cover.

### REFERENCES

Adami H-O, Bergström R, Persson I and Sparén P (1990) The incidence of ovarian cancer in Sweden, 1960–1984. *Am J Epidemiol* **132**: 446–452

Ahlbom A (1990) Some notes on brain tumor epidemiology. In *Trends in Cancer Mortality in Industrial Countries*, Davis DL and Hoel D (eds). *Ann NY Acad Sci* **609**: 179–185. New York Academy of Sciences: New York

Banatvala N, Mayo K, Megraud F, Jennings R, Deeks JJ and Feldman RA (1993) The cohort effect and *Helicobacter pylori*. *J Infectious Dis* **168**: 219–221

Banks PM (1992) Changes in diagnosis of non-Hodgkin's lymphoma over time. *Cancer Res* **52** (suppl.): 5453S–5455S

Barnes N, Cartwright RA, O'Brien C, Richards IDG, Roberts B and Bird CC (1986) Rising incidence of lymphoid malignancies – true or false? *Br J Cancer* **53**: 393–398

Beatson GT (1896) On the treatment of inoperable cases of carcinoma of the mamma: suggestions for a new method of treatment, with illustrative cases. *Lancet* **ii**: 104–107

Beral V (1974) Cancer of the cervix. A sexually transmitted infection? *Lancet* **i**: 1037–1040

Beral V, Hermon C, Muñoz N and Devesa SS (1994) Cervical cancer. In *Trends in Cancer Incidence and Mortality. Cancer Surveys*, Vols 19/20, Doll R, Fraumeni JF Jr and Muir CS (eds), pp. 265–285. Cold Harbor Laboratory Press: New York

Bergström R, Adami H-O, Möhner M, Zatonski W, Storm H, Ekbom A, Tretli S, Teppo L, Akre O and Hakulinen T (1996) Increase in testicular cancer incidence in six European countries: a birth cohort phenomenon. *J Natl Cancer Inst* **88**: 727–733

Black RJ, Sharp L and Kendrick SW (1993) *Trends in Cancer Survival in Scotland, 1968–90: Information and Statistics Division*. NHS in Scotland: Edinburgh

Blot WJ, Devesa SS, Kneller RW and Fraumeni JF Jr (1991) Rising incidence of adenocarcinoma of the esophagus and gastric cardia. *J Am Med Assoc* **265**: 1287–1289

Blot WJ, Devesa SS, McLaughlin JK and Fraumeni JF Jr (1994) Oral and pharyngeal cancers. In *Trends in Cancer Incidence and Mortality. Cancer Surveys*, Vols 19/20, Doll R, Fraumeni JF Jr and Muir CS (eds), pp. 23–42. Cold Spring Harbor Laboratory Press: New York

Brewster D, Crichton J and Muir C (1994) How accurate are Scottish cancer registration data? *Br J Cancer* **70**: 954–959

Cameron HM and McGoogan E (1981*a*) A prospective study of 1152 hospital autopsies: I. Inaccuracies in death certification. *J Pathol* **133**: 273–283

Cameron HM and McGoogan E (1981*b*) A prospective study of 1152 hospital autopsies: II. Analysis of inaccuracies in clinical diagnoses and their significance. *J Pathol* **133**: 285–300

Case RAM (1956) Cohort analysis of mortality rates as an historical or narrative technique. *Br J Prev Soc Med* **10**: 159–171

Coleman MP, Estève J, Damiecki P, Arslan A and Renard H (1993) *Trends in Cancer Incidence and Mortality*. IARC Scientific Publication no. 121. IARC: Lyon

Cuzick J (1994) Multiple myeloma. In *Trends in Cancer Incidence and Mortality. Cancer Surveys*, Vols 19/20, Doll R, Fraumeni JF Jr and Muir CS (eds), pp. 455–474. Cold Spring Harbor Laboratory Press: New York

Darby SC and Doll R (1987) Fallout, radiation doses near Dounreay and childhood leukaemia. *Br Med J* **294**: 603–607

Day NE and Varghese C (1994) Oesophageal cancer. In *Trends in Cancer Incidence and Mortality. Cancer Surveys*, Vols 19/20, Doll R, Fraumeni JF Jr and Muir CS (eds), pp. 43–54. Cold Spring Harbor Laboratory Press: New York

Desmeules M, Mikkelsen T and Mao Y (1992) Increasing incidence of primary malignant brain tumors: influence of diagnostic methods. *J Natl Cancer Inst* **84**: 442–445

Devesa SS and Fears T (1992) Non-Hodgkin's lymphoma time trends: United States and international data. *Cancer Res* **52** (suppl.): 5432S–5440S

Division of Epidemiology (1976) *Serial Mortality Tables. Neoplastic Diseases Volume 4. Scotland, 1911–70*. Division of Epidemiology, Institute of Cancer Research: London

Doll R (1990) Are we winning the fight against cancer? An epidemiological assessment. EACR-Mühlbock memorial lecture. *Eur J Cancer* **26**: 500–508

Dos Santos Silva I and Swerdlow AJ (1995) Recent trends in incidence of and mortality from breast, ovarian and endometrial cancers in England and Wales and their relation to changing fertility and oral contraceptive use. *Br J Cancer* **72**: 485–492

Draper GJ, Kroll ME and Stiller CA (1994) Childhood cancer. In *Trends in Cancer Incidence and Mortality. Cancer Surveys*, Vol 19/20. Doll R, Fraumeni JF Jr and Muir CS (eds), pp. 493–517. Cold Spring Harbor Laboratory Press: New York

Duguid HLD, Duncan ID and Currie J (1985) Screening for cervical intraepithelial neoplasia in Dundee and Angus 1962–81 and its relation with invasive cervical cancer. *Lancet* **ii**: 1053–1056

Early Breast Cancer Trialists' Collaborative Group (1992) Systemic treatment of early breast cancer by hormonal, cytotoxic, or immune therapy: 133 randomised trials involving 31 000 recurrences and 24 000 deaths among 75 000 women. *Lancet* **339**: 1–15, 71–85

Forman D, Pike MC, Davey G, Dawson S, Baker K, Chilvers CED, Oliver RTD and Coupland CAC (1994) Aetiology of testicular cancer: association with congenital abnormalities, age at puberty, infertility, and exercise. *Br Med J* **308**: 1393–1399

Gilliland FD and Samet JM (1994) Lung cancer. In *Trends in Cancer Incidence and Mortality. Cancer Surveys*, Vols 19/20, Doll R, Fraumeni JF Jr and Muir CS (eds), pp. 175–195. Cold Spring Harbor Laboratory Press: New York

Glass S, Gray M, Eden OB and Hann I (1987) Scottish validation study of cancer registration data childhood leukaemia 1968–1981 – I. *Leukemia Res* **11**: 881–885

Grulich AE, Swerdlow AJ, dos Santos Silva I and Beral V (1995) Is the apparent rise in cancer mortality in the elderly real? Analysis of changes in certification and coding of cause of death in England and Wales, 1970–1990. *Int J Cancer* **63**: 164–168

Hakulinen T, Teppo L and Saxén E (1978) Cancer of the eye, a review of trends and differentials. *World Health Stats Quarterly* **31**: 143–158

Hasle H and Mellemgaard A (1993) Hodgkin's disease diagnosed post mortem: a population based study. *Br J Cancer* **67**: 185–189

Hughes JS, Shaw KB and O'Riordan MC (1989) Radiation exposure of the UK population – 1988 review. NRPB R227. NRPB: Chilton, Oxfordshire

Information Services Division for the Scottish Health Service (1977) *Scottish Health Statistics, 1975*. HMSO: Edinburgh

Information Services Division for the Scottish Health Service (1982) *Scottish Health Statistics 1980*. HMSO: Edinburgh

Information and Statistics Division for the Scottish Health Service (1989) *Scottish Health Statistics 1989*. ISD: Edinburgh

Information and Statistics Division for the Scottish Health Service (1991) *Scottish Health Statistics 1991*. ISD: Edinburgh

Information and Statistics Division, National Health Service in Scotland (1993) *Scottish Health Statistics, 1993*. ISD: Edinburgh

Information and Statistics Division, Scottish Health Service (1997) *Births in Scotland 1976–1995*. ISD: Edinburgh

Kemp I, Boyle P, Smans M and Muir C (eds) (1985) *Atlas of Cancer in Scotland 1975–1980. Incidence and Epidemiological Perspective*. IARC Scientific Publication no. 72. IARC: Lyon

Kinlen LJ (1994) Leukaemia. In *Trends in Cancer Incidence and Mortality. Cancer Surveys*, Vols 19/20, Doll R, Fraumeni JF Jr and Muir CS (eds), pp. 475–491. Cold Spring Harbor Laboratory Press: New York

Kinlen LJ, O'Brien F, Clarke K, Balkwill A and Matthews F (1993) Rural population mixing and childhood leukaemia: effects of the North Sea oil industry in Scotland, including the area near Dounreay nuclear site. *Br Med J* **306**: 743–748

Kurihara M, Aoki K and Hisamichi S (1989) *Cancer Mortality Statistics in the World, 1950–1985*. University of Nagoya Press: Nagoya

La Vecchia C, Lucchini F, Negri E, Boyle P, Maisonneuve P and Levi F (1992) Trends of cancer mortality in Europe, 1955–1989. *Eur J Cancer* **28**: 132–235, 514–599; **28A**: 927–998, 1210–1281, 1509–1581

Lee PN (1976) *Statistics of Smoking in the United Kingdom. Research Paper 1*. 7th edn. Tobacco Research Council: London

Levi F, La Vecchia C, Lucchini F, Negri E and Boyle P (1992) Patterns of childhood cancer incidence and mortality in Europe. *Eur J Cancer* **28A**: 2028–2049

Linos A, Kyle RA, O'Fallon WM and Kurland LT (1981) Incidence and secular trend of multiple myeloma in Olmsted County, Minnesota: 1965–77. *J Natl Cancer Inst* **66**: 17–20

Lynch CF, Platz CE, Jones MP and Gazzaniga JM (1991) Cancer registry problems in classifying invasive bladder cancer. *J Natl Cancer Inst* **83**: 429–433

MacGregor JE, Moss SM, Parkin DM and Day NE (1985) A case–control study of cervical cancer screening in North East Scotland. *Br Med J* **290**: 1543–1546

MacKie RM and Hole DJ (1996) Incidence and thickness of primary tumours and survival of patients with cutaneous malignant melanoma in relation to socioeconomic status. *Br Med J* **312**: 1125–1128

MacKie RM, Hunter JAA, Aitchison TC, Hole D, McLaren K, Rankin R, Blessing K, Evans AT, Hutcheon AW, Jones DH, Soutar DS, Watson ACH, Cornbleet

MA and Smyth JF for the Scottish Melanoma Group (1992) Cutaneous malignant melanoma, Scotland, 1979–89. *Lancet* **339**: 971–975
Matheson LM, Dunnigan MG, Hole D and Gillis CR (1985) Incidence of colo-rectal, breast and lung cancer in a Scottish Asian population. *Health Bull* **43**: 245–249
Ministry of Agriculture, Fisheries and Food (1955). *Domestic Food Consumption and Expenditure, 1953. Annual Report of the National Food Survey Committee*. HMSO: London
Ministry of Agriculture, Fisheries and Food (1971) *Household Food Consumption and Expenditure: 1969. Annual Report of the National Food Survey Committee*. HMSO: London
Ministry of Agriculture, Fisheries and Food (1986) *Household Food Consumption and Expenditure: 1984. Annual Report of the National Food Survey Committee*. HMSO: London
Ministry of Agriculture, Fisheries and Food (1991) *Household Food Consumption and Expenditure, 1990, With a Study of Trends over the Period 1940–1990. Annual Report of the National Food Survey Committee*. HMSO: London
Ministry of Agriculture, Fisheries and Food (1996). *National Food Survey 1995*. The Stationery Office: London
Muir CS (1992) Classification. In *Cancer Incidence in Five Continents*, Vol. VI, Parkin DM, Muir CS, Whelan SL, Gao YT, Ferlay J and Powell J (eds), pp. 25–30. IARC Scientific Publication no 120. IARC: Lyon
Muir CS, Storm HH and Polednak A (1994) Brain and other nervous system tumours. In *Trends in Cancer Incidence and Mortality. Cancer Surveys*, Vols 19/20, Doll R, Fraumeni JF Jr and Muir CS (eds), pp. 369–392. Cold Spring Harbor Laboratory Press: New York
National Radiological Protection Board (1995) *Board Statement on Effects of Ultraviolet Radiation on Human Health, and Health Effects from Ultraviolet Radiation, Report of an Advisory Group on Non-Ionising Radiation*. NRPB: Chilton, Oxfordshire
Norris W (1820) A case of fungoid disease. *Edinburgh Med Surg J* **16**: 562–565
Office of Population Censuses and Surveys (1983) *Mortality Statistics: Comparison of 8th and 9th Revisions of the International Classification of Diseases, 1978 (sample)*. Series DH1, no. 10. HMSO: London
Office of Population Censuses and Surveys (1985). *Mortality Statistics, Cause, 1984*. Series DH2, no. 11. HMSO: London
Parkin DM, Muir CS, Whelan SL, Gao YT, Ferlay J and Powell J (eds) (1992) *Cancer Incidence in Five Continents*, Vol. VI. IARC Scientific Publication no. 120. International Agency for Research on Cancer: Lyon
Peto J, Hodgson JT, Matthews FE and Jones JR (1995) Continuing increase in mesothelioma mortality in Britain. *Lancet* **345**: 535–539
Potosky AL, Kessler L, Gridley G, Brown CC and Horm JW (1990) Rise in prostatic cancer associated with increased use of transurethral resection. *J Natl Cancer Inst* **82**: 1624–1628
Registrar General in Scotland (1904) *Forty-Seventh Detailed Annual Report of the Registrar General of Births, Deaths, and Marriages in Scotland (Abstracts of 1901)*. HMSO: Glasgow
Registrar General for Scotland (1914) *Fifty-Seventh Annual Report of the Registrar-General for Scotland 1911*. HMSO: Glasgow
Registrar General for Scotland (1931) *Seventy-Sixth Annual Report of the Registrar-General for Scotland, 1930*. HMSO: Edinburgh
Registrar-General for Scotland (1937) *Eighty-Second Annual Report of the Registrar-General for Scotland, 1936*. HMSO: Edinburgh
Registrar General for Scotland (1942) *Eighty-Sixth Annual Report of the Registrar-General for Scotland, 1940*. GRO: Edinburgh
Registrar General for Scotland (1961) *Annual Report of the Registrar-General for Scotland 1960*. No 106, HMSO: Edinburgh
Registrar General Scotland (1991) *Annual Report 1990*. GRO: Edinburgh
Registrar General Scotland (1996) *Annual Report 1995*. GRO: Edinburgh
Ries LAG (1994) Colorectal cancer survival. *J Natl Cancer Inst* **86**: 415
Saxén EA (1982) Trends: facts or fallacy. In *Trends in Cancer Incidence. Causes and Practical Implications*, Magnus K. (ed). Hemisphere Publishing: Washington
Scottish Council on Alcohol (1997) *UK Alcohol Statistics 1997*. Scottish Council on Alcohol: Glasgow
Sharpe RM and Skakkebaek NE (1993) Are oestrogens involved in falling sperm counts and disorders of the male reproductive tract? *Lancet* **341**: 1392–1395
Silverman DT, Morrison AS and Devesa SS (1996) Bladder cancer. In *Cancer Epidemiology and Prevention*, 2nd edn, Schottenfeld D and Fraumeni JF Jr (eds), pp. 1156–1179. Oxford University Press: New York
Smans M, Muir CS and Boyle P (eds) (1992) *Atlas of Cancer Mortality in the European Economic Community*. IARC Scientific Publication no. 107. IARC: Lyon
Spring JA and Buss DH (1977) Three centuries of alcohol in the British diet. *Nature* **270**: 567–572
Stephen AM and Sieber GM (1994) Trends in individual fat consumption in the UK 1900–1985. *Br J Nutrition* **71**: 775–788
Stewart HJ for the Scottish Cancer Trials Breast Group (1992) The Scottish trial of adjuvant tamoxifen in node-negative breast cancer. *J Natl Cancer Inst Monogr* **18**: 117–120
Swerdlow AJ (1989) Interpretation of England and Wales cancer mortality data: the effect of enquiries to certifiers for further information. *Br J Cancer* **59**: 787–791
Swerdlow AJ (1990) Effectiveness of primary prevention of occupational exposures on cancer risk. In *Evaluating Effectiveness of Primary Prevention of Cancer*, Hakama M, Beral V, Cullen JW and Parkin DM (eds), pp 23–56. IARC Scientific Publication no 103, IARC: Lyon
Swerdlow AJ (1997) Epidemiology of testicular cancer. In *Principles and Practice of Genitourinary Oncology*, Raghavan D, Scher HI, Leibel SA and Lange PH (eds), pp. 643–652. Lippincott-Raven: Philadelphia
Tomatis L, Aitio A, Day NE, Heseltine E, Kaldor J, Miller AB, Parkin DM and Riboli E (eds) (1990) *Cancer: Causes, Occurrence and Control*. IARC Scientific Publication no 100. IARC: Lyon
Turesson I, Zettervall O, Cuzick J, Waldenstrom JG and Velez R (1984) Comparison of trends in the incidence of multiple myeloma in Malmö, Sweden, and other countries, 1950–1979. *N Engl J Med* **310**: 421–424
Ursin G, Bernstein L and Pike MC (1994) Breast cancer. In *Trends in Cancer Incidence and Mortality. Cancer Surveys*, Vols 19/20. Doll R, Fraumeni JF Jr and Muir CS (eds), pp. 241–264. Cold Spring Harbor Laboratory Press: New York
van der Esch EP, Muir CS, Nectoux J, Macfarlane G, Maisonneuve P, Bharucha H, Briggs J, Cooke RA, Dempster AG, Essex WB, Hofer PA, Hood AF, Ironside P, Larsen TE, Little JH, Philipps R, Pfau RS, Prade M, Pozharisski KM, Rilke F and Schafler K (1991) Temporal change in diagnostic criteria as a cause of the increase of malignant melanoma over time is unlikely. *Int J Cancer* **47**: 483–490
Wald N, Kiryluk S, Darby S, Doll R, Pike M and Peto R (eds) (1988) *UK Smoking Statistics*. Oxford University Press: Oxford
World Health Organization (1978) *Manual of the International Statistical Classification of Diseases, Injuries, and Causes of Death*, Ninth Revision. WHO: Geneva

© Cancer Research Campaign 1998